# Urine‐Derived iPSC Neurospheres Uncover Proteomic Correlates of Clinical Severity in Dravet Syndrome

**DOI:** 10.1111/jnc.70452

**Published:** 2026-04-29

**Authors:** Michele Martins, Guillaume Nugue, Andrey Aguiar, Leticia R. Q. Souza, Paulo V. Abrantes, Julia Rodrigues Trajano, Mariana Stelling, Magno Junqueira, Stevens Rehen, Marília Zaluar P. Guimarães

**Affiliations:** ^1^ Departamento de Bioquímica, Instituto de Química Universidade Federal do Rio de Janeiro Rio de Janeiro Brazil; ^2^ Instituto D'Or de Pesquisa e Ensino (IDOR) Rio de Janeiro Brazil; ^3^ Instituto de Ciências Biomédicas Universidade Federal do Rio de Janeiro (UFRJ) Rio de Janeiro Brazil; ^4^ Instituto Federal do Rio de Janeiro Rio de Janeiro Brazil; ^5^ Departamento de Genética, Instituto de Biologia Universidade Federal do Rio de Janeiro (UFRJ) Rio de Janeiro Brazil

## Abstract

Dravet syndrome (DS) is a rare and severe childhood‐onset developmental epileptic encephalopathy caused primarily by mutations in the sodium channel gene SCN1A. Animal models have undeniably advanced our understanding of DS, but they still do not fully capture its clinical heterogeneity, highlighting the need for complementary human in vitro systems. Here, we generated induced pluripotent stem cells (iPSCs) from urine epithelial cells of three DS patients carrying distinct SCN1A variants and differentiated them into neural stem cells (NSCs) and early‐stage neurospheres. Clinical severity was assessed using the DANCE checklist, and molecular phenotypes were characterized through isobaric quantitative proteomics. Comparative analyses identified differences in protein abundance across patient‐derived lines, with distinct molecular patterns associated with clinical severity measures. The patient‐derived lines exhibited variability in protein groups related to synaptic organization, mitochondrial processes, and RNA processing, reflecting interindividual molecular differences within the cohort. These findings establish patient‐derived neurospheres as a scalable human model for investigating molecular variability in DS. This approach provides a framework to explore disease heterogeneity and provides a foundation for future studies linking molecular profiles to clinical variability in DS.

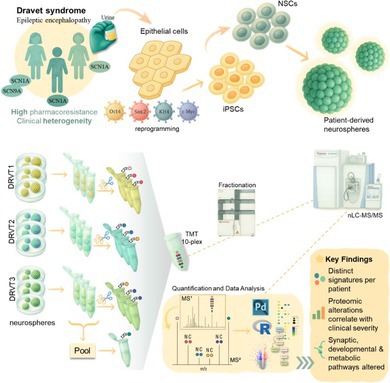

## Graphical abstract

Urine epithelial cells from Dravet syndrome (DS) patients carrying distinct SCN1A variants were reprogrammed into induced pluripotent stem cells (iPSCs) and differentiated into neural stem cells (NSCs) to generate patient‐derived neurospheres. Three neurosphere lines were analyzed in biological triplicates using isobaric quantitative proteomics. Peptides were labeled, combined, pre‐fractionated, and analyzed by LC–MS/MS, and protein abundances were processed in Proteome Discoverer and R for identification, quantification, and functional analysis. Comparative profiling revealed patient‐specific proteomic patterns and alterations correlating with clinical severity, involving synaptic, neurodevelopmental, and metabolic pathways.

**Abbreviations**
AFU: Arbitrary fluorescence units
ANOVA: Analysis of variance
DANCE: Dravet Syndrome Neurodevelopmental and Comorbidity Evaluation
DAPI: 4′,6‐diamidino‐2‐phenylindole
DS: Dravet Syndrome
FDR: False discovery rate
GFAP: Glial fibrillary acidic protein
GO: Gene Ontology
HSD: Honestly significant difference
iPSC: Induced pluripotent stem cell
KEGG: Kyoto Encyclopedia of Genes and Genomes
LC–MS/MS: Liquid chromatography–tandem mass spectrometry
Map 2: Microtubule‐associated protein 2
NSC: Neural stem cell
PAX6: Paired box protein 6
PLS‐DA: Partial Least Squares Discriminant Analysis
SCN1A: Sodium voltage‐gated channel alpha subunit 1
SD: Standard deviation
SEM: Standard error of the mean
TMT: Tandem mass tag
βIII‐tubulin (βTub): Beta III tubulin

## 1 Introduction

Dravet syndrome (DS), or severe myoclonic epilepsy of infancy, is a rare but devastating developmental epileptic encephalopathy with onset in the first year of life (Dravet et al. 2005). It is most commonly caused by pathogenic variants in the sodium channel gene SCN1A, leading to recurrent seizures, cognitive impairment, motor difficulties, and a high risk of premature death (Guerrini and Aicardi 2003; Catterall 2017). Despite advances in clinical care, DS remains highly refractory to treatment, and its complex pathophysiology is not fully understood.

Animal models of DS have provided crucial mechanistic insights, particularly regarding interneuron dysfunction and network hyperexcitability. They have shown that Nav1.1 channels, coded by SCN1A, are predominantly expressed in the central nervous system, especially in GABAergic hippocampal interneurons (Yu et al. 2006; Ogiwara et al. 2007; Cheah et al. 2012). Loss‐of‐function mutations in these models lead to altered neuronal firing and network hyperexcitability (Rubinstein et al. 2015). In particular, haploinsufficiency of *SCN1A* in parvalbumin‐positive GABAergic interneurons is sufficient to induce spontaneous and temperature‐evoked seizures, motor impairment, autism‐like behaviors, ataxia, and premature death—key features of DS (Yu et al. 2006; Ogiwara et al. 2007; Oakley et al. 2009; Cheah et al. 2012; Han et al. 2013).

However, species‐specific differences limit the relevance of these models, particularly for processes such as synaptic development, neuroinflammation, and metabolic regulation. To overcome these limitations, human induced pluripotent stem cells (iPSCs) have been widely used. Studies using DS patient‐derived iPSC neurons carrying *SCN1A* mutations have reported altered excitability in GABAergic (Higurashi et al. 2013; Sun et al. 2016; Kim 2017), glutamatergic (Jiao et al. 2013), or both neuronal populations (Liu et al. 2014). While informative, these monolayer (2D) systems often yield divergent results and do not capture the multicellular context of neural tissues (Akyuz et al. 2021).

More recently, 3D stem cell–based models such as brain organoids have been introduced to study complex neurodevelopmental disorders. These systems enable multicellular interactions and aspects of network organization to emerge, but they remain technically demanding, time‐consuming, and often variable across experiments. Neural spheroids (neurospheres) represent a pragmatic alternative; however, it is important to note that they correspond to early‐stage neurodevelopmental models and do not recapitulate mature neuronal circuits or fully developed synaptic networks. Instead, neurospheres preserve fundamental features of three‐dimensional tissue organization, are reproducible and scalable, and enable controlled comparisons across patient‐derived lines at comparable developmental stages.

DS is a developmental epileptic encephalopathy, in which pathological mechanisms extend beyond neuronal hyperexcitability to include disruptions in early neurodevelopmental programs, cellular stress responses, and, more recently, neuroinflammatory and neurodegenerative processes (Selvarajah et al. 2025). Accordingly, early‐stage iPSC‐derived models provide a relevant framework to investigate molecular pathways associated with initial phases of disease vulnerability, rather than mature network dysfunction.

To further explore the molecular landscape of DS, we performed quantitative tandem mass tag (TMT)‐based proteomic analysis. In human systems, organoids and neurospheres provide a more tissue‐relevant context than 2D cultures and enable the investigation of molecular processes associated with synaptic and cellular organization (Trujillo et al. 2019; Fair et al. 2020). Moreover, TMT‐based proteomic approaches have been used to characterize differentiation trajectories and metabolic remodeling in neural models and have recently been optimized for neurospheres, enabling reproducible comparisons across lines (Nugue et al. 2025; Park et al. 2023).

In this study, we generated early‐stage iPSC‐derived neurospheres from DS patients carrying distinct *SCN1A* variants and applied quantitative proteomics together with clinical severity assessment to determine whether these 3D models capture patient‐specific molecular differences and provide a translational framework to investigate disease heterogeneity.

## 2 Materials and Methods

### 2.1 Obtention of Somatic Cells From DS Patients

The study was approved by the Institutional Research Ethics Committee of IDOR and by the National Research Ethics Committee (protocol 67185123.2.0000.5249). Written informed consent was obtained from the patients' legal guardians prior to sample collection. Urine samples were obtained from patients clinically diagnosed with DS, and epithelial cells were isolated and cultured as previously described (Sochacki et al. 2016).

Patients differed in sex, age, and SCN1A/SCN9A genetic status (Table 1), reflecting interindividual variability within the cohort.

**TABLE 1:** Clinical and genetic characteristics of DS patient‐derived iPSC lines.

Cell line	Sex	Age at sample collection	Gene	Variant type	Zygosity	Variant (nucleotide)	Variant (protein)
DRVT1	Female	14	SCN1A	Deletion	Heterozygous	NM_006920.4: c.(694 + 1_695–1)_(1028 + 1_1029−1)*	—
DRVT2	Male	15	SCN1A	Nonsense	Heterozygous	NM_006920.4:c.1837C>T	NP_008851.3:p.(Arg613Ter)
DRVT3	Female	30	SCN1A	Deletion (fs)	Heterozygous	NM_006920.4:c.1472delC	NP_008851.3:p.(Lys492Argfs*52)
			SCN9A	Nonsense	Heterozygous	NM_002977.3:c.4843G>T	NP_002968.1:p.(Gly1615Ter)

### 2.2 iPSCs Generation and Neurosphere Culture

iPSCs were generated from epithelial cells isolated from the urine of DS patients using the CytoTune‐iPSCs 2.0 Sendai Reprogramming Kit (Thermo Fisher Scientific) according to the manufacturer's instructions. iPSCs were cultured in StemFlex medium (Thermo Fisher Scientific) on Matrigel‐coated plates (Corning) at 37°C and 5% CO_2_, with daily media changes to avoid spontaneous differentiation. Colonies were manually passaged at 70%–80% confluence. Pluripotency was confirmed by immunofluorescence staining and RT‐PCR analysis of pluripotency markers. Differentiation potential was assessed by embryoid body (EB) formation and detection of lineage‐specific markers from the three germ layers (Figure S1).

NSC induction was performed using PSC Neural Induction Medium (Thermo Fisher Scientific) on Geltrex LDEV‐Free–coated plates, following standard protocols. NSCs were expanded in Neural Expansion medium (1:1 Advanced DMEM/F12 and Neurobasal Medium, Thermo Fisher Scientific) supplemented with 1× neural induction supplement. For neurosphere formation, NSCs were dissociated with Accutase (Merck Millipore), centrifuged at 300 × g for 5 min, and resuspended in neural medium (1:1 DMEM/F12 and Neurobasal Medium, Thermo Fisher Scientific) supplemented with 1× N2 and 1× B27. Approximately 3 × 10^6^ NSCs per well were plated in 6‐well plates and cultured in suspension on an orbital shaker (90 rpm) for 7 days at 37°C and 5% CO_2_. Medium was changed every 3–4 days.

### 2.3 Immunofluorescence

Neurospheres were fixed in 4% paraformaldehyde (PFA) for 30 min, washed in PBS (3 × 5 min), and cryoprotected in 30% sucrose prior to embedding in OCT compound. Samples were frozen, sectioned at 10 μm using a cryostat, and mounted on glass slides. Sections from all three patient‐derived neurospheres were mounted on the same slide to reduce technical variability in immunofluorescence staining. Sections were then subjected to heat‐mediated antigen retrieval in sodium citrate buffer (pH 6.0), followed by permeabilization with 0.3% Triton X‐100 in PBS (PBT) for 15 min. Nonspecific binding was blocked using 3% BSA in PBT for 1 h, and samples were incubated with primary antibodies (Table 2) diluted in blocking solution overnight at 4°C in a humidified chamber. After washing in PBS, sections were incubated with appropriate fluorophore‐conjugated secondary antibodies (Table 2) for 2 h at room temperature. Nuclei were counterstained with DAPI, and samples were subsequently washed and mounted using Aqua‐Poly/Mount (Polysciences, 18606, USA). Slides were stored at 4°C in the dark until image acquisition.

**TABLE 2:** Antibodies.

Antibody	Supplier, cat#	Host species	Dilution
β‐tubulin III (TUJ1)	Neuromics, MO15013	Mouse	1:100
MAP2	Invitrogen, PA1‐10005	Chicken	1:10 000
PAX6	Thermo Fisher Scientific, 42‐6600	Rabbit	1:100
GFAP	Dako, Z0334	Mouse	1:100
Ki‐67	BD Pharmingen, 550 609	Mouse	1:100
Anti‐Rabbit IgG (H + L), Alexa Fluor Plus 647	Invitrogen, A32733TR	Goat	1:400
Anti‐Mouse IgG (H + L), Alexa Fluor 488	Invitrogen, A‐11008	Goat	1:400
Anti‐Chicken IgY (H + L), Alexa Fluor 594	Abcam, ab150172	Goat	1:400

Image acquisition was performed using the Cytation 5 cell imaging reader (BioTek Instruments, USA). Whole‐slide scans were performed at 1.25× magnification to define regions of interest (ROIs) containing several neurospheres, followed by imaging at 10× using appropriate filter sets. Acquisition parameters (illumination intensity, exposure time, and gain) were kept constant across samples. Z‐stacks (25–30 planes, 5 μm step) were acquired and projected using Gen5 software (BioTek Instruments, USA).

Quantitative analysis was performed using Gen5. Nuclei were segmented based on DAPI staining, and fluorescence intensity was measured per cell. Mean intensity values were calculated across all detected cells within each ROI. For area‐based measurements, marker‐positive area was normalized to DAPI‐positive area. Analysis parameters were kept constant across samples, and only signals above background were included.

Neurosphere counts per ROI were quantified using ImageJ following maximum intensity projection, thresholding, and particle analysis (≥ 2500 pixels). Representative images were processed for visualization using an unsharp mask filter without altering raw data used for quantification.

### 2.4 Protein Extraction and Digestion

After 7 days, neurospheres were collected, washed with cold PBS, and lysed in buffer containing 7 M urea, 2 M thiourea, 50 mM HEPES pH 8, 75 mM NaCl, 1 mM EDTA, and a complete protease inhibitor cocktail (Roche). Samples were homogenized by pipetting and sonicated for 10 min. Lysates were centrifuged at 10 000 × g for 10 min at 4°C, and the supernatant was collected for protein quantification using Qubit 2.0 (Invitrogen).

For digestion, 100 μg of protein per sample was reduced with TCEP (10 mM, 1 h, 30°C) and alkylated with iodoacetamide (IAA, 40 mM, 30 min, room temperature, in the dark). Samples were diluted 10× with 50 mM TEAB, and digestion was carried out with trypsin (1:50, w/w) overnight at 35°C. Digestion was stopped with 0.1% TFA (pH 2).

### 2.5 TMT Labeling and Peptide Fractionation

Resulting peptides were dried in a SpeedVac and resuspended in 100 mM TEAB. They were labeled with TMT 10‐plex reagents (Thermo Fisher Scientific) following the Martins et al. (2026) protocol. Briefly, each TMT condition included 10 μg of peptides in 5 μL of 50 mM HEPES (pH 8.5). We added TMT dissolved in ACN to reach a final concentration of 12 mM. The reaction was incubated for 1 h at room temperature. Specific conditions were assigned to each TMT channel as follows. Three DS patient‐derived lines were analyzed in biological triplicate (nine channels total); channel 131 was reserved for a pooled sample for quality control and potential batch correction. The TMT‐labeled peptide mixture was pre‐fractionated off‐line by basic pH reversed‐phase liquid chromatography prior to LC–MS/MS analysis. Samples were resuspended in 20 mM ammonium bicarbonate (AB) and injected into a Thermo UFLC system equipped with a Gemini 5 μm C18 110 Å, 250 × 4.60 mm column (Phenomenex), at a flow rate of 0.5 mL/min, using mobile phases A (20 mM AB) and B (20 mM AB with 90% acetonitrile, ACN). Fraction pools were generated based on peak separation and intensity. The pooled fractions were dried in a SpeedVac and reconstituted in 0.1% formic acid.

### 2.6 LC–MS/MS Analysis

Peptide samples were analyzed on a nanoLC easy‐1000 system coupled to a Q‐Exactive Plus mass spectrometer (Thermo Fisher Scientific) operating in data‐dependent acquisition (DDA) mode. The main parameters included a 5%–40% ACN linear gradient over 120 min, 2.5 kV spray voltage, 200°C capillary temperature, MS1 from 375 to 1800 m/z (resolution 70 000), MS2 with resolution 35 000, NCE 32, 2 m/z isolation window, and dynamic exclusion of 45 s.

### 2.7 Protein Identification and Quantification

Proteomics data were acquired from three independent biological replicates per cell line (separate neurosphere differentiation batches), distributed across two TMT‐10plex labeling batches. A common pooled‐sample reference channel was included in each batch to enable inter‐batch normalization via Internal Reference Scaling (IRS).

Raw spectra (.RAW files) were processed in Proteome Discoverer (version 3.2) using Sequest HT searched against the UniProt human proteome database. Search parameters included a precursor mass tolerance of 10 ppm, fragment tolerance of 0.02 Da, up to two missed cleavages, variable modifications (Met oxidation, +15.995 Da; TMT 10‐plex on N‐terminus and Lys, +229.163 Da), and fixed carbamidomethylation of Cys (+57.021 Da). Peptide and protein identifications were filtered at 1% FDR.

Downstream quantification and statistical analyses were performed in R. TMT reporter ion intensities were log_2_‐transformed and normalized using TMM followed by IRS batch correction (Plubell et al. 2017). Differential expression across the three DS lines (DRVT1, DRVT2, and DRVT3) was assessed by one‐way ANOVA with Tukey's HSD post hoc test for pairwise comparisons. Proteins were considered significantly differentially expressed at a Tukey‐adjusted *p*‐value < 0.05 and |log_2_FC| > 0.1, a conservative threshold chosen to account for the systematic ratio compression inherent to isobaric labeling (Ting et al. 2011; DOI: 10.1038/nmeth.1714). Gene Ontology, KEGG, and Reactome enrichment analyses were performed using clusterProfiler 4.0 (Wu et al. 2021).

### 2.8 Derivation of the DANCE‐Based Clinical Severity Score

The DANCE (Domains of Assessment of Neurocognitive and Clinical Endpoints) checklist was adapted from previous studies (Giorgi et al. 2024) and completed by caregivers of DS patients. Based on the responses, we developed a scoring table to quantify clinical severity per domain. The checklist comprises four main domains: Cognition and behavior, Motor abilities, Daily living, and Family quality of life. Since all four domains have a different number of questions to assess them, each mark on questions associated with core symptoms, comorbidities, and deleterious effect on quality of life in the DANCE checklist has been recorded and expressed as a ratio of maximum severity, which corresponds to full scoring in the respective domain.

For each domain, we summed the raw scores and then normalized the value obtained by the maximum domain score:

$${\text{DomainScore}}_{d}=\frac{\sum_{i=1}^{n}x_{1}}{\sum_{i=1}^{n}\mathrm{Ma}x_{1}}$$

This normalization allows domains with different numbers of questions to contribute equally to the overall score, where $x_{i}$ is the observed score for item i, ${\mathrm{Max}}_{i}$ is the maximum possible score for item i and n is the number of items in the domain. The Severity DANCE score for each patient was then obtained as the simple arithmetic mean of the four domain percentages:

$${\text{Severity}}_{\text{DANCE}}=\frac{\sum_{d=1}^{n}{\text{DomainScore}}_{d}}{4}$$

The score ranges were interpreted as follows: 0%–20% = very mild severity; 21%–40% = mild severity; 41%–60% = moderate severity; 61%–80% = high severity; and 81%–100% = very high severity. These values were used as a continuous clinical gradient to evaluate whether proteomic modules followed the same trend observed at the functional and behavioral level.

### 2.9 Integration of DANCE Score and Proteomic Data

To integrate molecular and clinical information, an ordinal score was generated for each functional module, reflecting the direction of pathway enrichment across lineages and oriented according to the clinical severity gradient derived from the DANCE questionnaire. Each module was coded as 2 (strongly enriched), 1 (enriched against one lineage), or 0 (absent/ambiguous). Modules expected to decrease with severity were assigned positive values, whereas those expected to increase with severity were assigned negative values. Neutral terms, such as extracellular matrix organization, were kept unchanged. These values were used to explore associations between proteomic patterns and clinical severity without implying causal relationships.

## 3 Results

### 3.1 Patients' Clinical and SCN1A Mutation Characterizations

Somatic cells were collected from urine samples of three DS patients. Diagnostic confirmation was established based on clinical presentation and detection of deleterious variants in SCN1A and SCN9A genes (Table 1). DRVT1 carried a heterozygous deletion encompassing exons 6 and 7 of SCN1A. DRVT2 presented a heterozygous nonsense mutation introducing a premature stop codon at amino acid position 613 of SCN1A, accompanied by a homozygous polymorphic variant (NM_006920.5:c.603‐91A, rs3812718, minor allele frequency ~0.48) previously associated with altered antiepileptic drug response (Tate et al. 2005; Thompson et al. 2011; Zhou et al. 2012a, 2012b; Margari et al. 2018). DRVT3 harbored a homozygous frameshift mutation at codon 492 of SCN1A and an additional nonsense mutation at codon 1625 of SCN9A.

To assess disease severity and associated comorbidities, we employed the DANCE checklist, a validated instrument for evaluating functional impairment in DS, translated to Brazilian Portuguese and completed by patient caregivers (Giorgi et al. 2024). Responses were categorized across four functional domains: cognition and behavior, motor abilities, daily living skills, and family quality of life. Details for each functional domain and their respective scores are summarized in Table S1.

**FIGURE 1:**
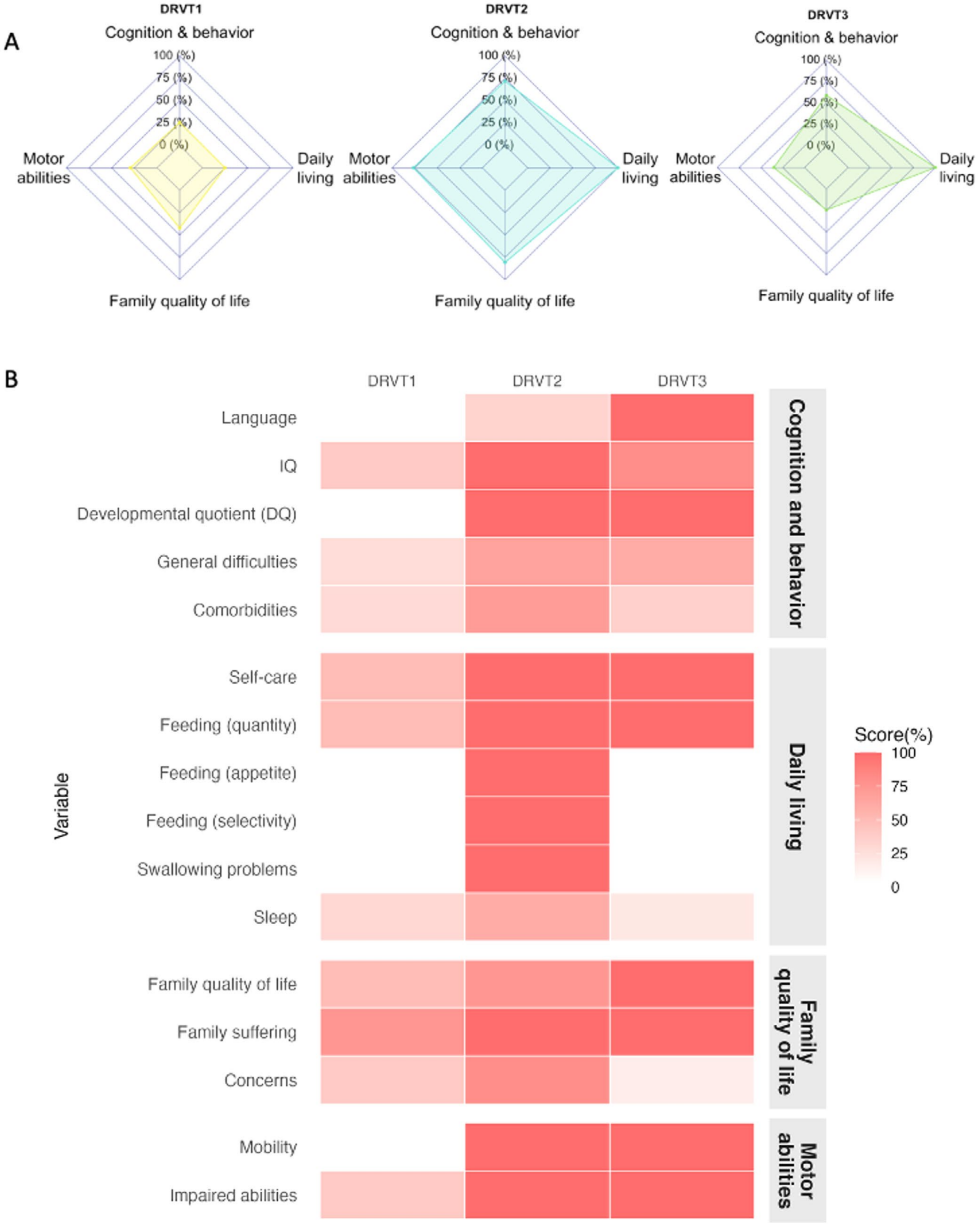
Clinical profiles of DS patients based on DANCE checklist responses. (A) Radar plots summarizing scores across four functional domains (cognition and behavior, daily living, motor abilities, and family quality of life). The higher percentages correspond to higher severity, so larger polygons indicate more impaired patients. (B) Heatmap displays item‐level scores, expressed as a percentage of each item.

This systematic evaluation revealed heterogeneity among the three patients. DRVT1 exhibited mild overall impairment (DANCE severity score: 31%), while DRVT2 demonstrated very high severity (82%) with pronounced deficits across cognition and behavior, motor function, and family quality of life. DRVT3 showed moderate severity (55%), primarily affecting cognitive and behavioral domains. Radar plots (Figure 1A) and the heatmap (Figure 1B) illustrate this gradient of clinical severity across the four functional domains assessed. The radar plots provide an integrated representation of each patient's functional status, where higher percentages reflect greater severity within each domain. Accordingly, the area enclosed by each polygon expands proportionally with increasing impairment, illustrating how functional deficits differ across domains. The heatmap (Figure 1B) shows the original item‐level scores normalized within each domain, highlighting intra‐domain variability and the most severely affected features for each patient.

The clinical severity scores derived from the DANCE questionnaire were consistent with independent objective clinical measures such as the intelligence quotient (IQ). Patients with greater cognitive impairment, DRVT2 (IQ < 20, profound intellectual disability) and DRVT3 (IQ 21–34, severe), also showed the highest DANCE severity scores, whereas DRVT1, classified as mild intellectual disability (IQ 50–69), presented the lowest score.

### 3.2 iPSCs, NSCs, and Neurospheres Exhibit Efficient and Comparable Differentiation Across Patient Lines

Patient‐derived hiPSCs were successfully generated from all three DS cases. The resulting cell lines formed stable colonies that maintained characteristic pluripotency features. Immunofluorescence staining and RT‐PCR analysis confirmed robust expression of endogenous pluripotency markers across all three lines. Complete absence of residual Sendai virus transgenes verified successful reprogramming. Selected clones demonstrated trilineage differentiation capacity, and low‐pass whole genome sequencing revealed preserved chromosomal integrity without detectable large‐scale copy number variations (Figures S1 and S2).

**FIGURE 2:**
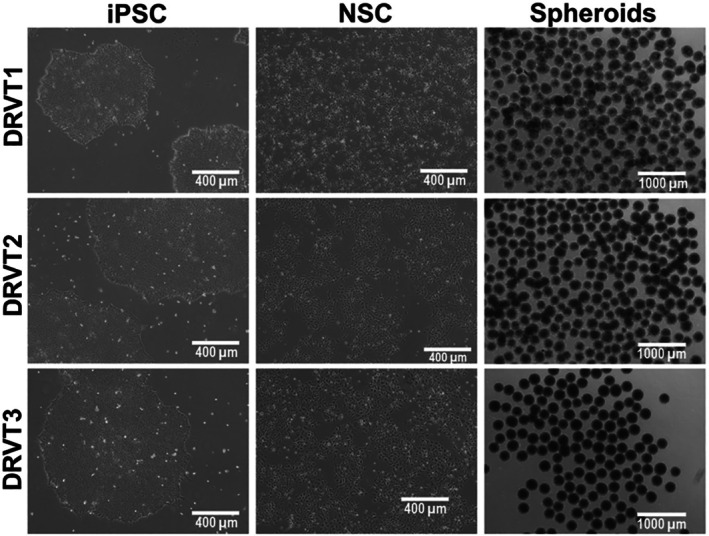
Morphological progression of DS patient–derived iPSCs into NSCs and spheroids. Representative bright‐field images of three independent DS iPSCs lines (DRVT1–3) at three differentiation stages: Induced pluripotent stem cells (iPSCs), neural stem cells (NSC), and neural spheroids. iPSCs formed compact colonies with clear borders (left panels), which differentiated into proliferative NSCs with elongated, neuroepithelial‐like morphology (middle panels). Upon aggregation, NSCs generated homogeneous spheroids with defined edges (right panels). Microphotographs were adjusted for brightness and contrast to improve homogeneity and visualization. Scale bars: 400 μm for iPSCs and NSCs, and 1000 μm for neurospheres.

Following neural induction protocols, all three iPSC lines efficiently differentiated into NSCs, which spontaneously organized into three‐dimensional neurospheres (Figure 2).

**FIGURE 3:**
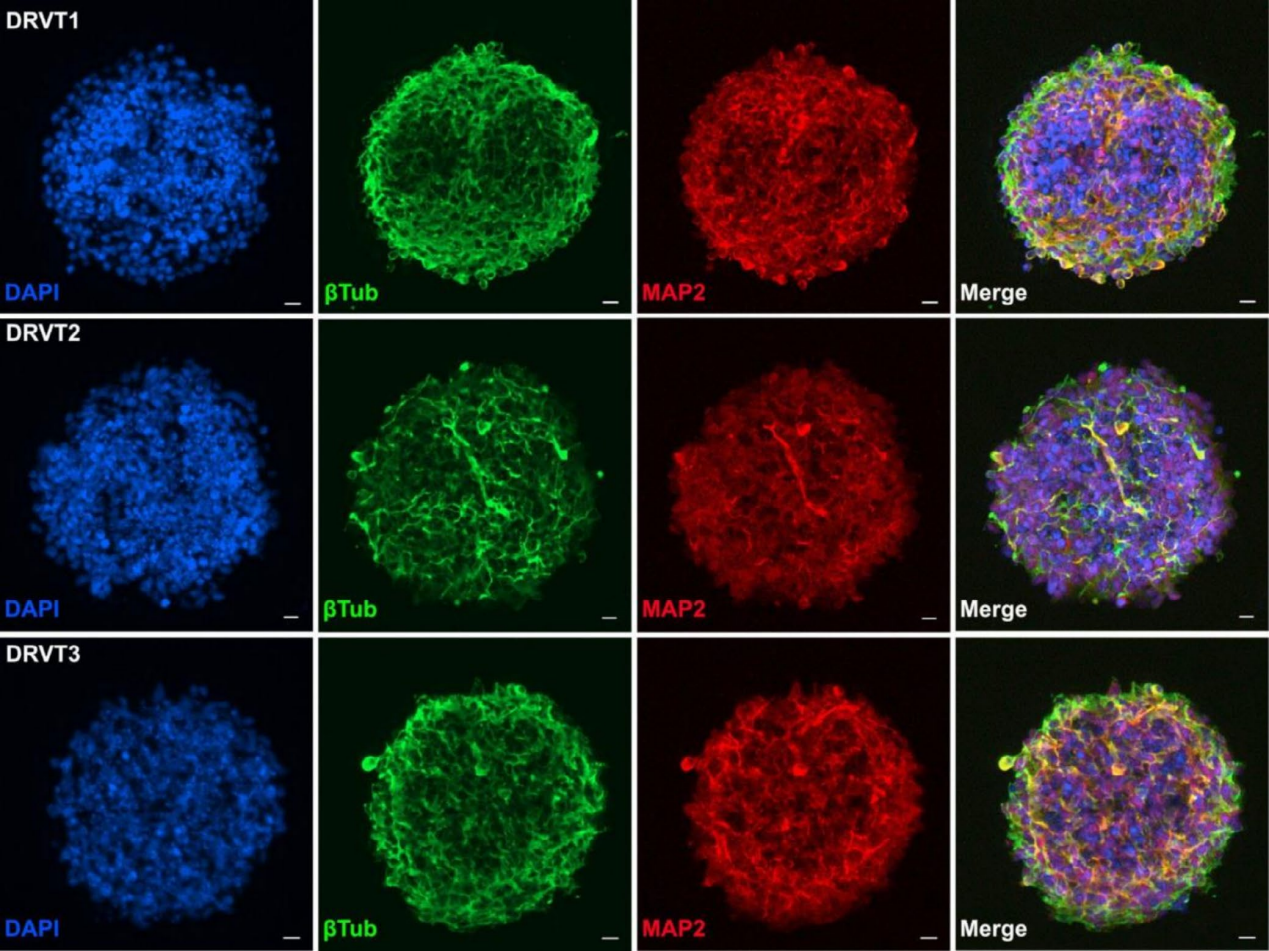
Immunofluorescence characterization of early‐stage patient‐derived neurospheres. Representative immunofluorescence images of neurospheres derived from three Dravet Syndrome (DS) Induced Pluripotent Stem Cell (iPSC) lines (DRVT1–3). Nuclei are labeled with DAPI (blue), βIII‐tubulin (βTub, green) marks early neuronal populations, and MAP2 (red) indicates neuronal differentiation. All lines show βIII‐tubulin–positive cells with MAP2 expression detectable throughout the spheroids. Scale bars: 10 μm.

To further characterize these cultures, we performed immunofluorescence analysis of neurospheres derived from all three patient lines (Figure 3; Figures S3 and S4). βIII‐tubulin–positive cells were detected throughout the spheroids, indicating the presence of early neuronal populations, while MAP2 expression was also observed, consistent with neuronal differentiation. Notably, the spatial distribution and morphology of MAP2 signal were diffuse and lacked organized dendritic structures, in line with an early developmental stage. Consistent with this, GFAP‐positive cells were detected, suggesting the presence of radial glia/astroglial lineage cells (Figure S3), while PAX6 staining confirmed the persistence of neural progenitor populations within the spheroids (Figure S4). Quantification of marker intensity and labeled area across the three lines further supported the presence of mixed neural populations with comparable lineage representation (Figure S5). Together, these observations indicate that the neurospheres correspond to early‐stage neural assemblies composed of progenitors and differentiating neurons, rather than mature neuronal networks, and provide a defined cellular context for the proteomic analyses performed in this study.

### 3.3 Proteomic Profiling Reveals Patient‐Specific Molecular Patterns

To investigate whether neurosphere models could capture patient‐specific molecular features, we performed comprehensive quantitative proteomics using TMT‐based across all three DS cell lines.

**FIGURE 4:**
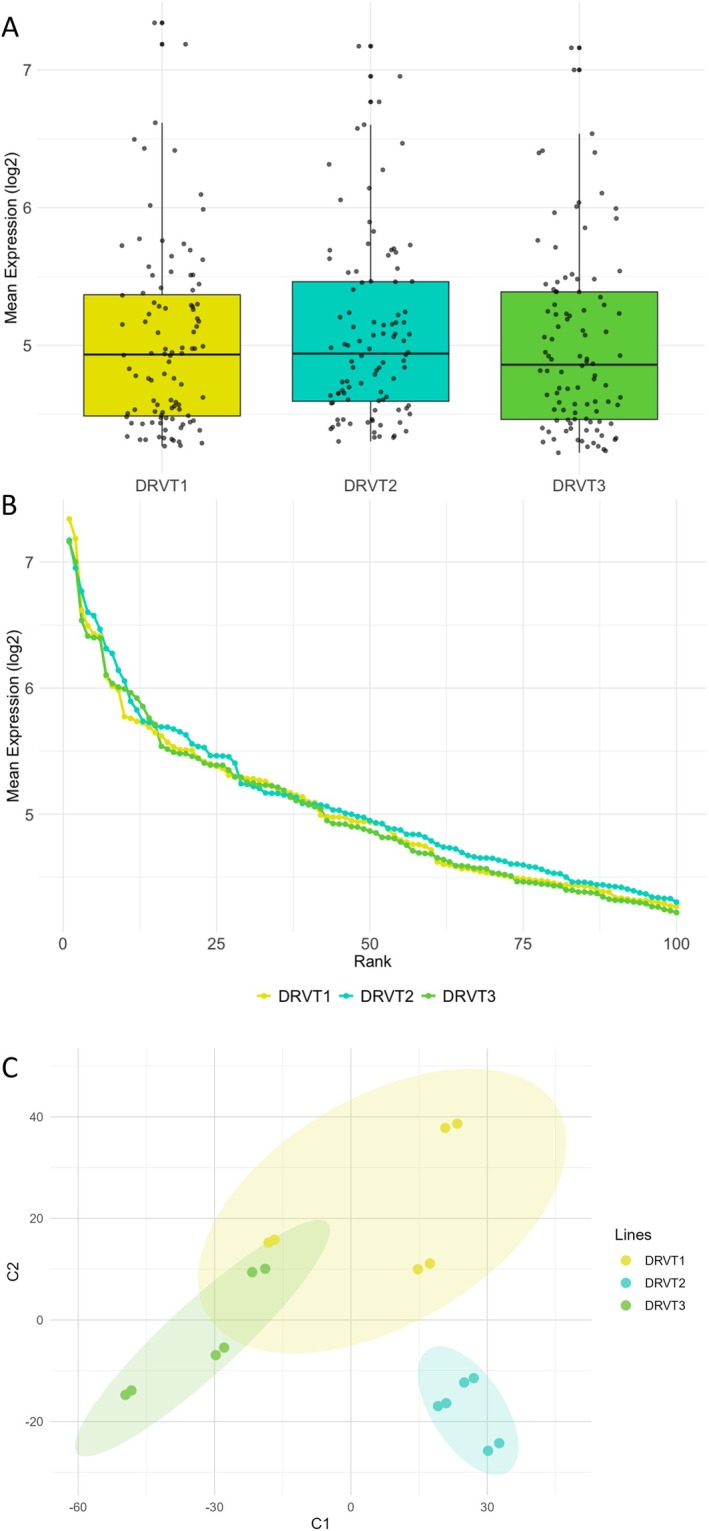
Protein expression profiles in three patient‐derived Dravet Syndrome (DS) cell lines. (A) Distribution of mean expression levels (log_2_) for the 100 most abundant proteins in each cell line. The central line indicates the median, and box limits represent the interquartile range; dots represent individual values. (B) Mean expression profiles (log_2_) of the proteins ranked by expression level for each cell line, showing overall protein abundance trends. (C) Partial Least Squares Discriminant Analysis (PLS‐DA) based on the relative abundance of proteins.

The three patient‐derived lines displayed comparable overall protein expression patterns (Figure 4). Distribution of mean log_2_ expression values for the 100 most abundant proteins showed consistency across DRVT1, DRVT2, and DRVT3 (Figure 4A), and protein ranking by abundance revealed similar trends among lines (Figure 4B). Although the overall abundance distributions were highly similar among the three lines, (PLS‐DA) we identified reproducible proteomic patterns that distinguish DRVT1, DRVT2, and DRVT3 (Figure 4C). This indicates that, despite sharing a common proteomic backbone, each line retains line‐specific features.

While all cell lines share the same panel of identified proteins, analysis of abundance profiles revealed variations among them. This variability was leveraged to define, for each lineage, a molecular profile based on relative protein quantification, providing the foundation for subsequent statistical and correlational analyses.

### 3.4 Differential Expression Identifies DRVT2 as a Molecular Outlier Driving Most Severity‐Associated Changes

**FIGURE 5:**
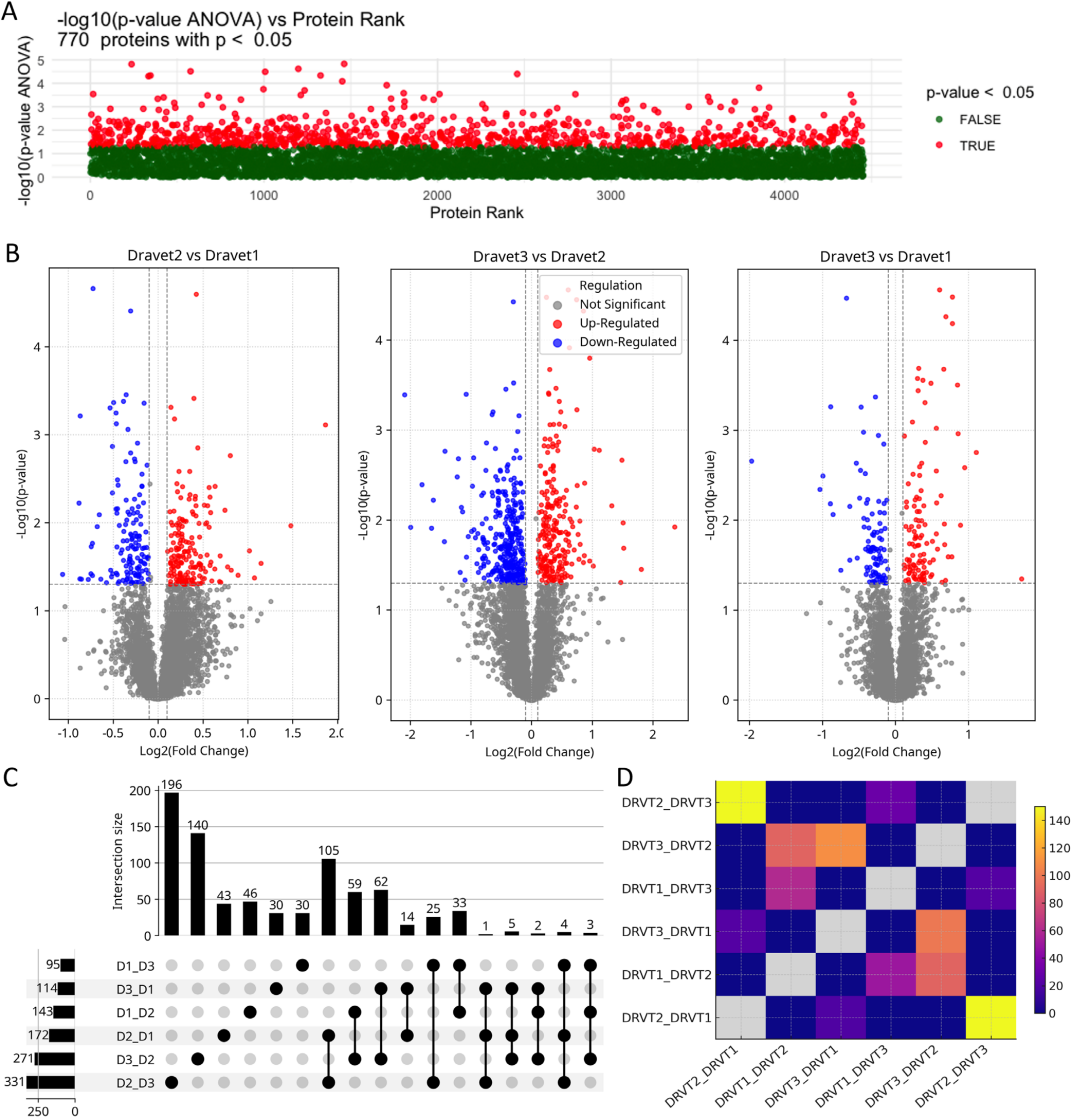
Proteomic variation and overlap across patient‐derived Dravet Syndrome (DS) neurosphere lines. (A) Statistical assessment of protein abundance variation across all samples. Each dot represents a quantified protein, with red indicating significant differences by ANOVA (*p* < 0.05, with 770 differential proteins). (B) Volcano plots of pairwise comparisons (DRVT1 vs. DRVT2, DRVT1 vs. DRVT3, and DRVT2 vs. DRVT3) based on Tukey's post hoc test. Red: Upregulated (log_2_FC > 0.1, *p* < 0.05); blue: Downregulated (log_2_FC < −0.1, *p* < 0.05); gray: Not significant. Dashed lines indicate fold‐change (±0.1) and *p*‐value (0.05) thresholds. (C) UpSet plot summarizing the intersection size among protein sets identified in each pairwise comparison. Horizontal bars represent the total number of proteins with significant differences per comparison, and vertical bars indicate the number of shared proteins across comparisons. (D) Heatmap of Fisher's exact test (−log_10_ *p*‐value) for the overlap between sets identified in pairwise comparisons. Warmer colors indicate greater statistical significance of overlap. Comparisons involving DRVT2 show higher overlap, whereas DRVT1 and DRVT3 exhibit lower shared protein sets.

Statistical analysis identified differences in protein abundance across the three patient‐derived lines. A parametric ANOVA test (*p* < 0.05) detected 770 proteins with significant variance among samples (Figure 5A). Pairwise comparisons using the Tukey HSD test were visualized using volcano plots (Figure 5B), illustrating the magnitude and direction of relative abundance differences between lines. Based on these comparisons, 307 proteins differed between DRVT1 and DRVT2, 203 between DRVT1 and DRVT3, and 543 between DRVT2 and DRVT3. These analyses are presented to describe interindividual variability within the cohort and are not intended to define disease‐associated changes.

The UpSet plot (Figure 5C) revealed an asymmetric distribution of differential proteins. DRVT2 showed upregulation of 331 and 172 proteins in comparison with DRVT1 and DRVT3, respectively, more than observed for the other two lines. The largest intersections (196, 140, 105, 62 proteins) involve comparisons with DRVT2, indicating this lineage as a molecular outlier. In contrast, the intersections between DRVT1 and DRVT3 did not exceed 59 proteins. The high number of differentially expressed proteins shared between the comparisons involving DRVT2 indicates that DRVT1 and DRVT3 share a pattern, with regulation opposite to that of DRVT2 for most of these targets.

Analysis of protein set intersections using UpSet plots, together with Fisher's exact test (Figure 5D), indicated significant overlap between protein sets identified in pairwise comparisons. Comparisons involving DRVT2 showed higher levels of overlap (−log_10_ *p*‐value > 90), whereas comparisons between DRVT1 and DRVT3 were lower (−log_10_ *p*‐value < 20). Notably, many of the shared proteins across comparisons involving DRVT2 displayed opposite directions of relative abundance when compared to DRVT1 and DRVT3. Consistent with this pattern, DRVT1 and DRVT3 exhibited more similar abundance profiles across a subset of proteins, while DRVT2 showed a comparatively distinct pattern within the cohort, under shared experimental conditions.

**FIGURE 6:**
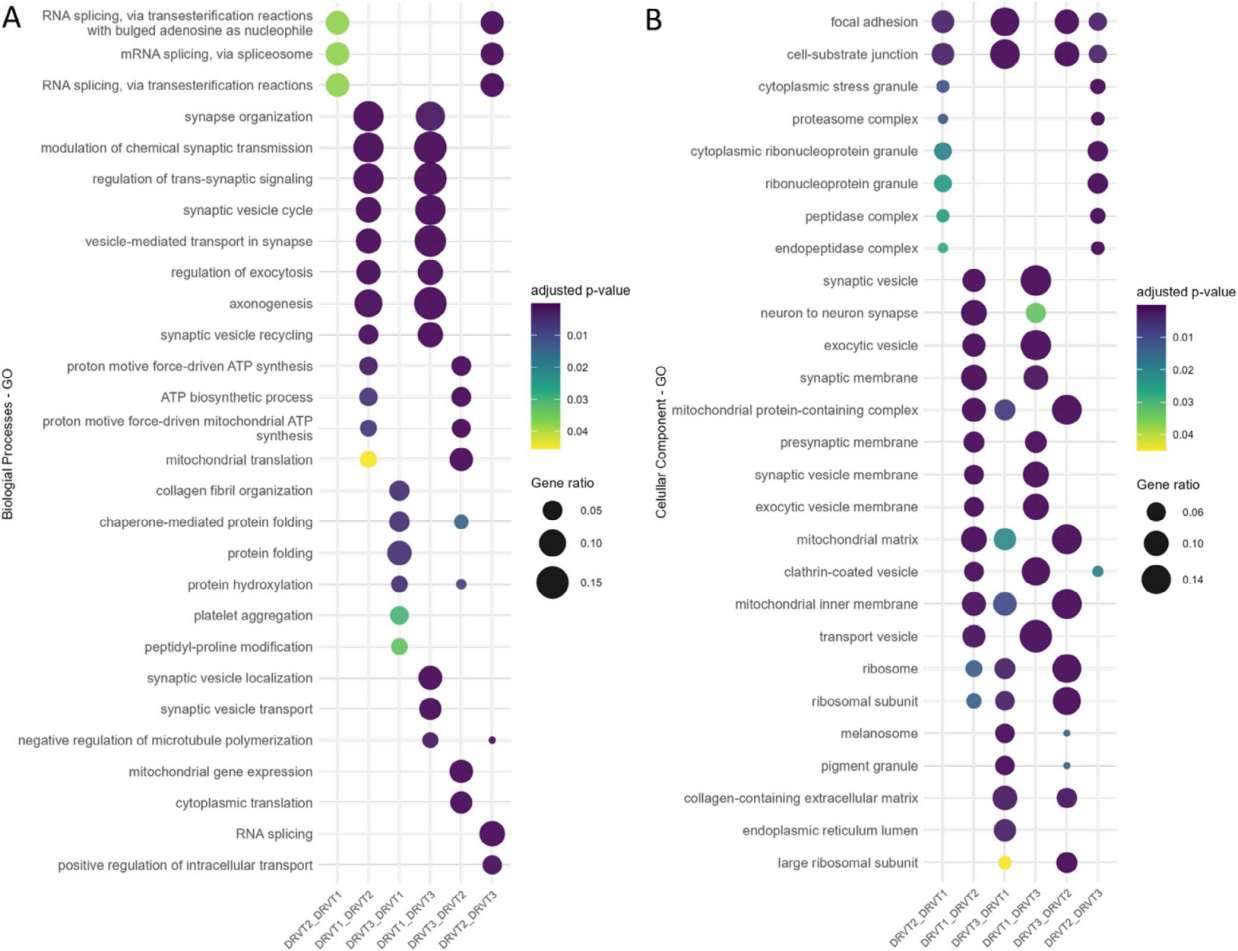
Dotplot by Gene Ontology (GO) enrichment of differentially expressed proteins in pairwise comparisons of Dravet Syndrome (DS) patient‐derived cell lines, where the first group in each comparison is upregulated relative to the second group. Dot size represents the number of associated proteins, and color scale indicates the adjusted *p*‐value (Benjamini–Hochberg correction). (A) Biological process terms. (B) Cellular component terms.

Functional enrichment analysis (EnrichGO) was performed via clusterProfiler to identify biological processes associated with patterns of protein abundance across patient‐derived lines (Figure 6). Among Gene Ontology (GO) Biological Process terms, proteins with higher relative abundance in DRVT1 compared to DRVT2 and DRVT3 were associated with processes related to synaptic formation and chemical transmission, including synapse organization, modulation of chemical synaptic transmission, synaptic vesicle cycle, vesicle‐mediated transport in synapse, regulation of exocytosis, and axonogenesis. In the Cellular Component category, proteins with higher relative abundance in DRVT1 were associated with terms such as synaptic vesicle, presynaptic membrane, synaptic membrane, and neuron‐to‐neuron synapse.

In contrast, proteins with lower abundance in DRVT2 compared to DRVT1 and DRVT3 were associated with processes related to mitochondrial bioenergetics, including mitochondrial translation, ATP biosynthetic process, oxidative phosphorylation and cristae formation. Additionally, proteins with higher relative abundance in DRVT2 were associated with terms related to RNA processing, including RNA splicing and ribonucleoprotein/stress granule‐related categories. These enrichments reflect differences in protein abundance profiles between patient‐derived lines and should be interpreted as associative rather than indicative of direct functional mechanisms.

**FIGURE 7:**
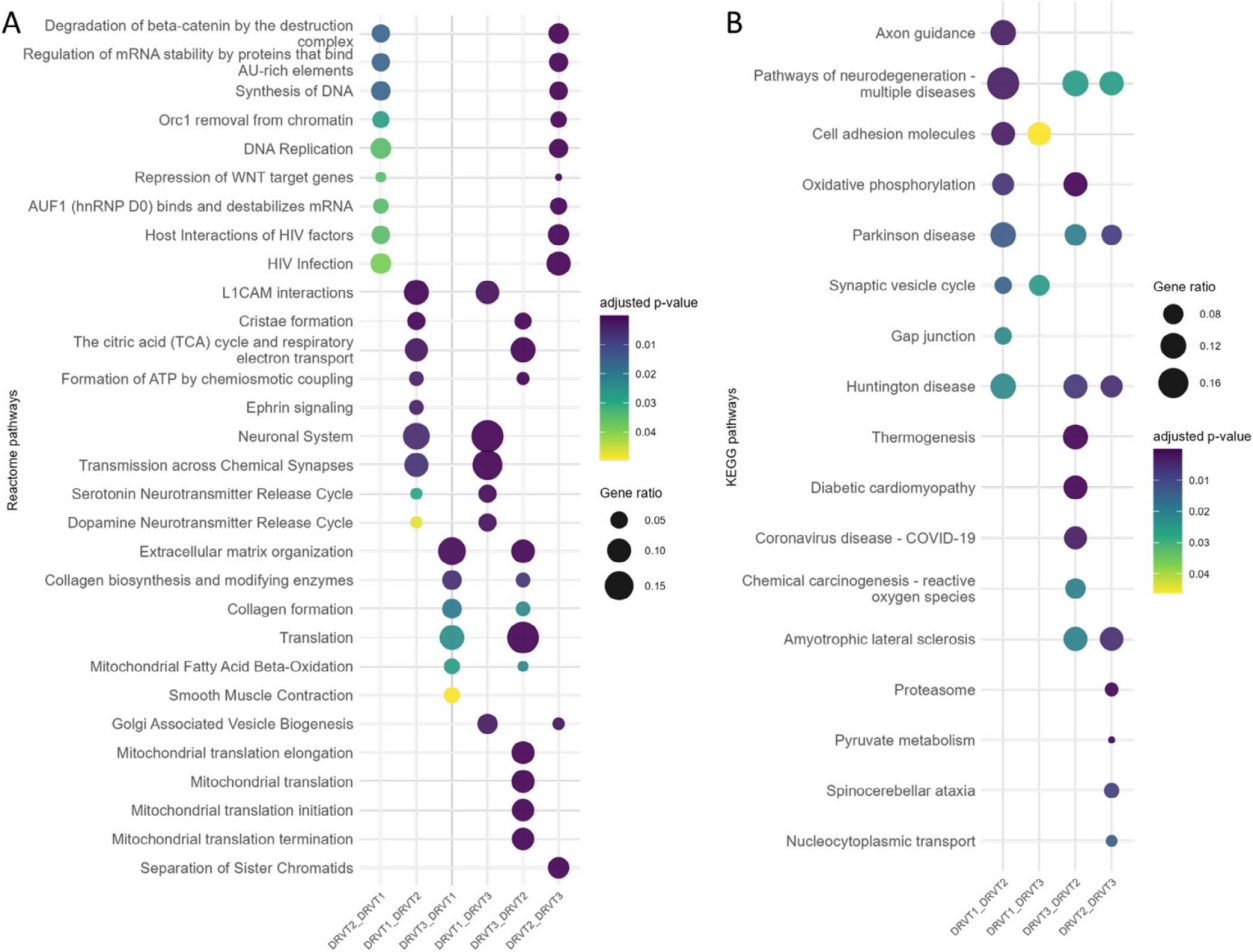
Dotplot by pathway enrichment of differentially expressed proteins in pairwise comparisons of Dravet Syndrome (DS) patient‐derived cell lines, where the first group in each comparison is upregulated relative to the second group. Dot size represents the number of associated proteins, and color scale indicates the adjusted *p*‐value (Benjamini–Hochberg correction). (A) Reactome pathway terms. (B) Kyoto Encyclopedia of Genes and Genomes (KEGG) pathway terms.

Reactome and KEGG pathway analyses corroborated these findings (Figure 7). Terms including cristae formation, TCA cycle and respiratory electron transport, ATP synthesis by chemiosmotic coupling, oxidative phosphorylation, and thermogenesis were upregulated in DRVT1 and DRVT3 relative to DRVT2. Pathways related to neuronal systems, synaptic vesicle cycle, serotonergic and dopaminergic neurotransmitter release, axon guidance, and L1CAM interactions showed lower abundance in DRVT2 and DRVT3 compared with DRVT1, consistent with GO enrichment patterns.

### 3.5 Proteomic Modules Track With the DANCE Clinical Gradient and Reflect the Continuum of Disease Severity

**FIGURE 8:**
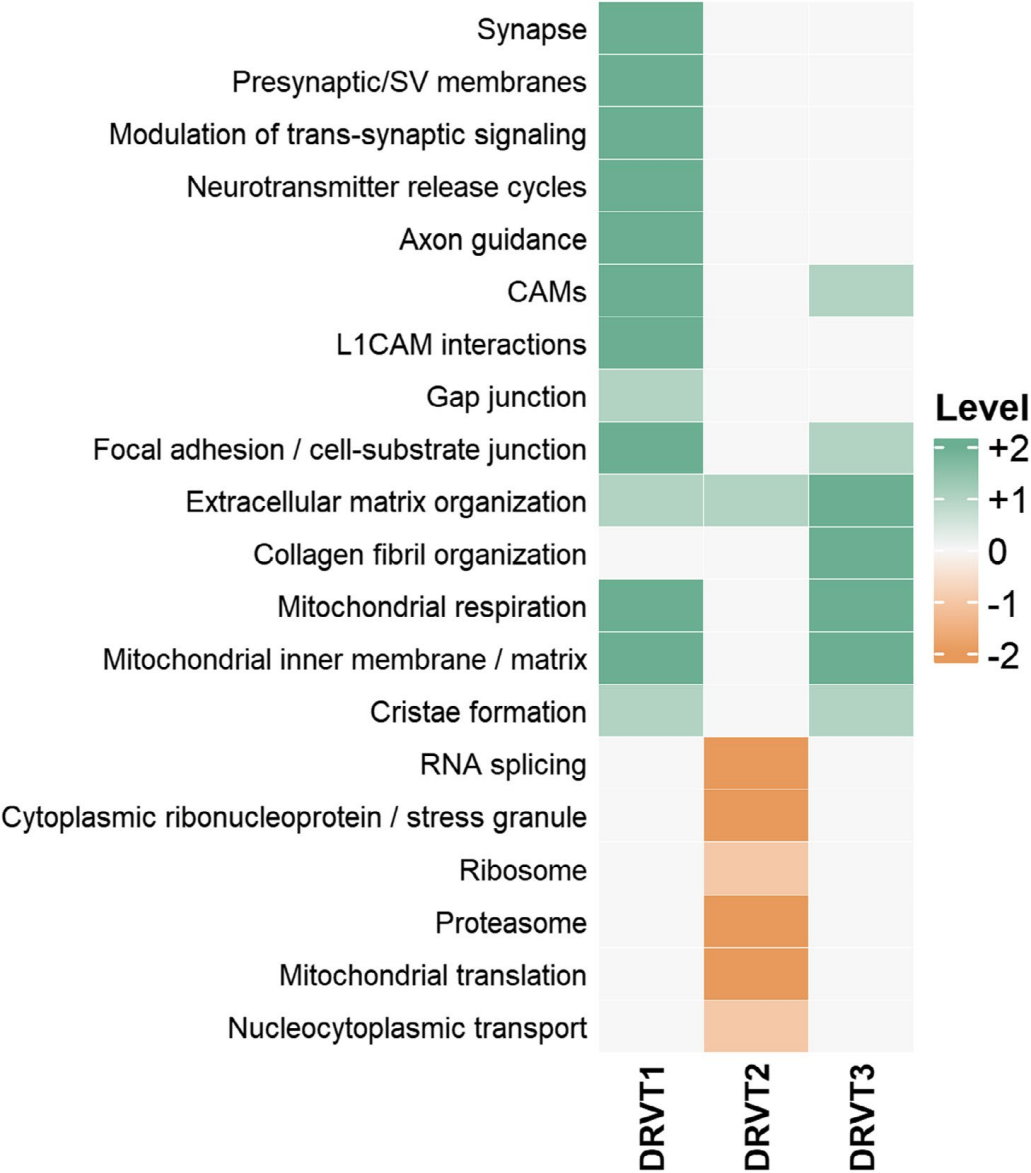
Heatmap of processes/pathway‐level scores. Positive values indicate overexpressed modules that are linked to lower severity, and negative values indicate overexpressed modules that are linked to higher severity. See Table S3 for more information.

To summarize these pathway‐level differences relative to clinical data, the ordinal module scores were compared to the DANCE severity gradient (DRVT1 = 31 < DRVT3 = 55 < DRVT2 = 82). In the matrix (Figure 8), positive values indicate modules aligned with lower severity, whereas negative values indicate modules aligned with higher severity. The correspondence between modules and DANCE scores is detailed in Table S3. Given *n* = 3 donors, this visualization is descriptive and does not constitute a statistical association test.

This representation revealed that most biological processes qualitatively followed the clinical severity gradient. Synaptic, adhesion, and respiratory modules aligned with milder phenotypes, whereas posttranscriptional and proteostatic modules correlated with greater impairment. This pattern indicates that the proteomic landscape of patient‐derived neurospheres reflects clinically relevant aspects of disease severity.

### 3.6 Synaptic and Neurotransmission Pathways Show Graded Impairment Consistent With Clinical Severity

**FIGURE 9:**
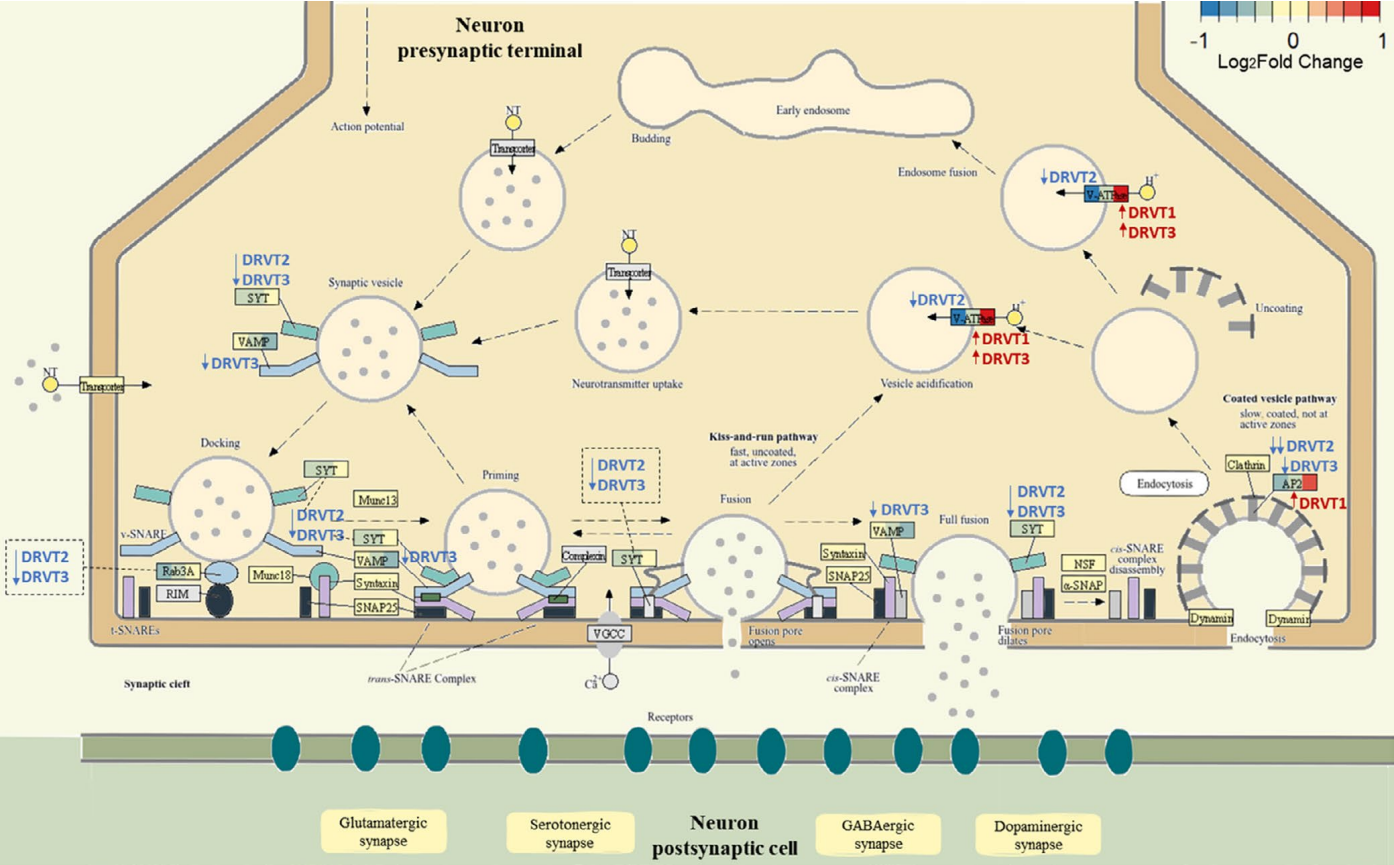
Mapping of differentially abundant proteins onto the synaptic vesicle cycle pathway (hsa04721—KEGG) and associated neurotransmitter systems. Arrows indicate the direction and regulation of change for each comparison, proteins in yellow are identified, and blue and red colored shading corresponds to Log_2_ fold change values. Related neurotransmitter systems GABAergic (hsa04727), glutamatergic (hsa04724), serotonergic (hsa04726), and dopaminergic (hsa04728) synapses mapped are shown in Figures S3–S6.

Systematically, we mapped the differentially abundant proteins into KEGG synaptic pathways. To specifically explore neurotransmitter systems, four systems were mapped: the GABAergic, glutamatergic, serotonergic, and dopaminergic synapses. The synaptic vesicle cycle (hsa04721) was the central pathway, representing the core process of neurotransmitter release across neurochemically defined synapses, and was included in the main analysis (Figure 9), common to all neurochemically defined synapses (Figure 9; detailed maps in Figures S6–S9).

Multiple proteins central to the synaptic vesicle cycle were identified, providing broad coverage of the main steps involved in synaptic transmission. Both DRVT2 and DRVT3 showed reduced levels of components associated with vesicle release compared with DRVT1. Several of these differentially abundant proteins participate in neurotransmitter release and synaptic plasticity, and selected targets are discussed below in the context of synaptic alterations related to DS.

## 4 Discussion

In this study, we established iPSCs‐derived neurospheres from three Dravet syndrome (DS) patients carrying distinct SCN1A variants and performed quantitative proteomic profiling to explore molecular patterns associated with clinical severity. Immunofluorescence characterization and quantitative analyses indicated that all three lines exhibit comparable representation of neural progenitor, glial, and neuronal populations, supporting the use of this system for inter‐patient comparisons while minimizing confounding effects from differences in lineage composition. Our results show that patient‐derived 3D neural models can capture inter‐patient molecular variability within DS and that these differences are associated with clinical severity measures.

Previous iPSC‐based studies of DS have mainly relied on neuronal cultures directed toward specific cell types, including GABAergic, glutamatergic, or dopaminergic neurons. while these approaches provided important insights into how SCN1A mutations may alter neuronal excitability, They have often yielded divergent findings, with some studies reporting preferential effects on GABAergic neurons (Higurashi et al. 2013; Sun et al. 2016; Kim 2017), others on glutamatergic neurons (Jiao et al. 2013), and others on both neuronal populations (Liu et al. 2014) or dopaminergic cells (Maeda 2016). These discrepancies highlight limitations of reductionist 2D systems for studying a disorder that likely involves multiple interacting cellular and molecular processes. Other studies have also modeled DS using brain organoids derived from iPSCs. Zayat et al. (2023) generated patient‐derived ventral forebrain organoids and demonstrated preserved differentiation potential and regional marker expression, supporting their use as DS models. Complementing this, Yokoi et al. (2023) employed DS brain organoids to assess neuronal network activity, and reported hyperexcitability and altered responses to antiepileptic drugs. In this context, our study contributes a complementary approach by examining proteomic variability in a scalable early‐stage 3D neural model and relating these molecular patterns to clinical heterogeneity.

The patient‐derived lines analyzed here reflect interindividual genetic variability within DS, each carrying a distinct heterozygous loss‐of‐function *SCN1A* variant: a multi‐exon deletion (DRVT1), a nonsense variant p.(Arg613Ter) (DRVT2), and a frameshift deletion p.(Lys492Argfs*52) (DRVT3). In addition, DRVT3 carries a heterozygous nonsense variant in *SCN9A*, which has been proposed as a potential genetic modifier in epilepsy and DS (Singh et al. 2009). Such genomic differences may contribute to distinct proteomic patterns across patient‐derived lines.

Proteins with higher relative abundance in DRVT1 than in DRVT2 and DRVT3 were enriched for synaptic and neurotransmission‐related GO terms. This pattern is consistent with prior observations in SCN1A models implicating excitatory–inhibitory imbalance and synaptic remodeling (Uchino et al. 2021), as well as hippocampal proteomic alterations involving synaptic transmission and plasticity in Scn1a models (Miljanovic et al. 2021). In the present study, these enrichment patterns may indicate relative preservation of proteins associated with synaptic organization in DRVT1 compared with the other lines, although no direct functional conclusions can be drawn from the current data.

In contrast, proteins associated with mitochondrial bioenergetic processes showed lower relative abundance in DRVT2 than in DRVT1 and DRVT3. Previous studies in DS have reported mitochondrial abnormalities, including alterations in respiratory chain complexes and bioenergetic pathways (Craig et al. 2012; Doccini et al. 2015; Figueroa et al. 2025; Kumar et al. 2016). Our findings are consistent with this literature at the level of pathway association, but they should not be interpreted as direct evidence of mitochondrial dysfunction, as no functional metabolic assays were performed. Similarly, Reactome and KEGG enrichment analyses identified mitochondrial and synaptic‐related pathways that differed across lines, suggesting distinct molecular organization of bioenergetic and neuronal protein groups within the cohort.

Beyond mitochondrial‐associated pathways, DRVT2 also showed enrichment of proteins related to RNA metabolism and cellular stress responses, including RNA splicing and ribonucleoprotein/stress granule categories. Variants affecting SCN1A splicing have been implicated in DS and other epilepsies (Wang et al. 2012; Sparber et al. 2024; Zhang et al. 2024), and antisense oligonucleotide approaches targeting aberrant splicing have shown promise in preclinical DS models (Yuan et al. 2024). In addition, proteins such as G3BP1, which were more abundant in DRVT2 than in DRVT1 and DRVT3, are involved in stress granule assembly and have been associated with neurodevelopmental disorders that include epilepsy (Jia et al. 2022). In our dataset, these enrichments may reflect differences in posttranscriptional and stress‐response pathways across patient‐derived lines.

Several mapped proteins within synaptic pathways also differed across lines. In the synaptic vesicle cycle, AP2S1, ATP6V0D1, and ATP6V0C showed lower relative abundance in DRVT2. These proteins have been implicated in vesicle trafficking and acidification processes relevant to neurotransmitter handling (Toei et al. 2010; Gu et al. 2013), and variants in ATP6V0C have been associated with neurodevelopmental phenotypes including epilepsy (Mattison et al. 2023). Although these observations do not establish functional impairment, they further support the presence of patient‐specific differences in proteins associated with synaptic pathways.

Alterations in GNAO1 and GNAI1 were also observed across mapped neurotransmitter‐related pathways. Variants in these genes have been associated with developmental delay, hypotonia, movement disorders, and epilepsy (Saitsu et al. 2016; Feng et al. 2018; Muir et al. 2021). In the GABAergic synapse, PLCL1 showed higher relative abundance in DRVT2; previous work has linked this gene to seizure susceptibility and TrkB‐related signaling (Kim et al. 2019; Gu et al. 2015). In the glutamatergic pathway, lower GRM5 abundance in DRVT2, together with relatively higher NCDN abundance, may indicate differences in proteins associated with glutamatergic signaling and receptor modulation (Wang et al. 2009; Ojha et al. 2022). In the serotonergic pathway, lower abundance of G‐protein subunits and CASP3 in DRVT2 and DRVT3 suggests additional differences in proteins linked to receptor‐associated signaling and seizure‐related pathways (Henshall et al. 2000; Baculis et al. 2017). Likewise, proteins mapped to the dopaminergic synapse, including CAMK2A, KIF5C, and PPP3CA, were reduced in some comparisons and are notable because variants or reduced function in these genes have been associated with epileptic phenotypes and neurodevelopmental disorders (Küry et al. 2017; Chia et al. 2018; Qian et al. 2018; Banerjee et al. 2024).

A recurring observation in our dataset was that DRVT2, the patient line with the highest clinical severity score in the present cohort, showed a proteomic profile distinct from DRVT1 and DRVT3, with differences involving mitochondrial, RNA‐processing, and synaptic‐associated proteins. By contrast, DRVT1, the least severe line in our cohort, showed relative enrichment for proteins associated with synaptic organization, vesicle cycling, and axon‐related terms. These findings suggest that proteomic profiling in patient‐derived neurospheres may capture molecular variability that parallels clinical heterogeneity within DS. However, these associations should be interpreted cautiously, as the present study was not designed to establish causal or predictive relationships between molecular features and clinical severity.

Overall, the differential abundance patterns observed across neurotransmitter systems, synaptic‐associated pathways, and bioenergetic processes support the view that patient‐derived neurospheres can preserve interindividual molecular patterns within DS. Rather than defining disease‐specific mechanisms, these data provide an initial framework for investigating how patient‐level molecular variation may relate to differences in clinical presentation.

Importantly, the absence of neurotypical control neurosphere or iPSC lines limits the ability to interpret these findings in the context of disease‐specific mechanisms, and therefore conclusions regarding pathogenic processes in DS should be made with caution.

## 5 Conclusions

Our findings indicate that patient‐derived neurospheres can reflect inter‐patient molecular differences within DS and that proteomic profiles vary in association with clinical severity measures in this cohort. These results support the rationale for further investigation toward a molecular framework for patient stratification. The integration of proteomic data with the DANCE‐derived clinical severity score highlighted pathways associated with neuronal signaling, RNA processing, and mitochondrial‐related processes that varied across lines. More broadly, these results suggest that early‐stage patient‐derived neurospheres may be useful for studying clinical heterogeneity in severe developmental epileptic encephalopathies.

However, the absence of neurotypical control lines limits the interpretation of these findings in the context of disease‐specific mechanisms, and this should be considered when extrapolating conclusions regarding DS pathophysiology.

### 5.1 Limitations and Future Directions

This study has some limitations. First, the number of patient‐derived lines analyzed was small, reflecting the rarity of DS and the complexity of generating iPSC‐based models. Larger cohorts will be necessary to validate the proteomic patterns identified here and to determine whether they generalize across different SCN1A variants and genetic backgrounds. Second, the absence of neurotypical control neurosphere or iPSC lines limits the ability to interpret the observed proteomic patterns in the context of disease‐specific mechanisms. While the study was designed to investigate interindividual variability within DS, the lack of a control group precludes direct comparisons with neurotypical molecular baselines and should be considered when interpreting associations with pathogenic processes. Third, while proteomic profiling revealed pathways associated with clinical severity, functional validation is needed to determine whether these molecular differences contribute to disease phenotypes. Electrophysiological recordings, metabolic flux assays, and targeted perturbations, including pharmacological modulation of mitochondrial pathways or antisense oligonucleotides targeting SCN1A splicing, would provide important mechanistic insight. Fourth, our neurospheres represent early neurodevelopmental stages and lack the full cellular complexity of mature brain tissue, including microglia and vasculature, which may also influence disease mechanisms. Finally, sex represents an additional source of biological variability in this cohort, and its contribution to the observed proteomic differences cannot be disentangled in the present study.

Future studies should integrate multi‐omics approaches, including transcriptomics and metabolomics, with functional assays to provide a more comprehensive view of DS biology. In addition, testing therapeutic interventions directly in patient‐derived neurospheres may help accelerate the translation of these findings into personalized medicine strategies. Expanding these models to include larger numbers of patient‐derived lines and, where possible, multiple clones per patient will be important for defining robust molecular patterns associated with clinical heterogeneity in DS.

In conclusion, our study shows that patient‐derived neurospheres can reveal molecular patterns associated with clinical severity in DS. By linking proteomic profiling with patient‐level clinical data, this platform may contribute to future efforts in biomarker discovery and patient stratification in developmental epileptic encephalopathies.

## Author Contributions

**Julia Rodrigues Trajano:** methodology, writing – review and editing, formal analysis. **Guillaume Nugue:** methodology, writing – review and editing, formal analysis, visualization, conceptualization, data curation. **Michele Martins:** formal analysis, visualization, conceptualization, writing – original draft, writing – review and editing, methodology, data curation. **Leticia R. Q. Souza:** methodology, writing – review and editing. **Andrey Aguiar:** conceptualization, methodology, writing – review and editing. **Paulo V. Abrantes:** methodology, writing – review and editing, formal analysis. **Mariana Stelling:** methodology, writing – review and editing. **Stevens Rehen:** project administration, funding acquisition, writing – review and editing. **Magno Junqueira:** funding acquisition, writing – review and editing, project administration. **Marília Zaluar P. Guimarães:** conceptualization, funding acquisition, writing – original draft, writing – review and editing, visualization, project administration.

## Funding

This project was supported by the Coordenação de Aperfeiçoamento de Pessoal de Nível Superior (CAPES), the Fundação Carlos Chagas Filho de Amparo à Pesquisa do Estado do Rio de Janeiro (FAPERJ), the Conselho Nacional de Desenvolvimento Científico e Tecnológico (CNPq), Financiadora de Estudos e Projetos (FINEP) and PRONAS from Ministério da Saúde.

## Ethics Statement

All procedures involving human participants were conducted in accordance with the ethical standards of the institutional and national research committees. The study was approved by the Institutional Research Ethics Committee of IDOR and by the Brazilian National Research Ethics Committee (protocol no. 67185123.2.0000.5249). Written informed consent was obtained from the patients' legal guardians prior to sample collection.

## Conflicts of Interest

The authors declare no conflicts of interest.

## Supporting information


**Table S1:** Clinical severity scores from the DS‐Associated Neuropsychiatric Comorbidities Evaluation (DANCE) checklist applied to Dravet syndrome (DS) patients. Values represent composite severity scores (0%–100%; higher = worse). Individual item scores were normalized as percentages of their maximum value, then averaged within each domain to obtain domain scores. The global severity score represents the mean across all four domains.
**Table S2:** Primer sequences.
**Table S3:** Summary of functional module enrichment across Dravet patient‐derived neurosphere lineages and DANCE checklist.
**Table S4:** TMT‐10plex sample assignment and batch design. Each of the two labeling batches contained nine biological samples from three Dravet syndrome iPSC‐derived neurosphere lines (DRAVET1, DRAVET2, DRAVET3; three independent differentiation replicates each) and one pooled reference sample (channel 131) used for Internal Reference Scaling (IRS) inter‐batch normalization.
**Figure S1:** Characterization of Induced Pluripotent Stem Cells (iPSCs) generated from Dravet syndrome patients. (A) Immunofluorescence staining of the pluripotency markers OCT‐3/4, SOX2, NANOG, TRA 1‐60, TRA‐1‐81 and SSEA‐4. DAPI shows nuclei counterstaining in blue. Scale bar: 100 μm. (B) Silencing of Sendai reprogramming factors was confirmed by RT‐PCR. (C) Germ layer markers transcripts of iPSCs cells differentiated into embryoid bodies; endoderm marker alpha‐feto protein (AFP), mesoderm marker homeobox protein MSX‐1 and ectoderm marker paired box gene 6 (PAX6) by RT‐PCR. Glyceraldehyde 3 phosphate dehydrogenase (GAPDH) was used as internal control.
**Figure S2:** Aneuploidy analysis of Induced Pluripotent Stem Cells (iPSCs) using low‐pass whole genome sequencing. Chromosome copy number analysis was carried out using low‐pass whole genome sequencing. Diagrams are a snapshot of IGV Light Whole Genome View screen depicting overviews of the cell lines diploid chromosome sets. Dots correspond to sequencing tiles approximately 2 Mb long. (A) DRVT‐1: MAPD (median absolute pairwise difference): 0.129; read count: 459214; total number of bases: 88.7 Mb; total number of bases (AQ20): 78.6 Mb; % bases (AQ20): 88.6; mean coverage depth(fold): 0.0286. (B) DRVT‐2: MAPD: 0.128; read count: 604906; total number of bases: 122 Mb; total number of bases (AQ20): 108 Mb; % bases (AQ20): 88.5; mean coverage depth(fold): 0.04. (C) DRVT‐3: MAPD: 0.129; read count: 612418; total number of bases: 113 Mb; total number of bases (AQ20): 99 Mb; % bases (AQ20): 87.6; mean coverage depth(fold): 0.0.
**Figure S3:** Immunofluorescence detection of GFAP and MAP2 in patient‐derived neurospheres. Representative immunofluorescence images of neurospheres derived from three Dravet syndrome iPSC lines (DRVT1‐3). Nuclei are labeled with DAPI (blue), GFAP (green) marks radial glia/astroglial lineage cells, and MAP2 (red) indicates neuronal differentiation. GFAP‐positive cells are distributed throughout the spheroids, consistent with the presence of glial‐lineage cells at early developmental stages. MAP2 signal is detectable but displays diffuse morphology without organized dendritic structures. Merged images illustrate the spatial distribution of these cell populations within the neurospheres. Scale bars: 10 μm.
**Figure S4:** Immunofluorescence detection of PAX6 and MAP2 in patient‐derived neurospheres. Representative immunofluorescence images of neurospheres derived from three Dravet syndrome iPSC lines (DRVT1‐3). Nuclei are labeled with DAPI (blue), PAX6 (green) marks neural progenitor populations, and MAP2 (red) indicates neuronal differentiation. PAX6‐positive cells are observed across the spheroids, supporting the persistence of neural progenitors at this stage. MAP2 expression is present but exhibits diffuse distribution, consistent with early neuronal differentiation. Merged images show co‐distribution of progenitor and neuronal markers. Scale bars: 10 μm.
**Figure S5:** Quantification of lineage marker expression in patient‐derived neurospheres. (A) Mean fluorescence intensity (arbitrary fluorescence units, AFU) for βIII ‐tubulin (βTub), MAP2, GFAP, and PAX6 across neurospheres derived from three Dravet syndrome iPSC lines (DRVT1‐3). Bars represent mean values for each line, and individual data points correspond to independent measurements. (B) Quantification of labeled area normalized to DAPI area for βIII‐tubulin (βTub), MAP2, GFAP, and PAX6 across the same neurospheres. Bars represent mean values, and individual data points correspond to independent differentiation of neurospheres. Error bars represent SEM. For each differentiation (*n* = 2), between 20 and 215 neurospheres were analyzed. Together, these analyses indicate the presence of neuronal (βIII‐tubulin, MAP2), glial (GFAP), and progenitor (PAX6) markers across all lines, consistent with mixed neural populations at an early developmental stage.
**Figure S6:** KEGG mapping of the glutamatergic synapse (hsa04724) in Dravet patient–derived lines.
**Figure S7:** KEGG mapping of the serotonergic synapse (hsa04726) in Dravet patient–derived lines.
**Figure S8:** KEGG mapping of the GABAergic synapse (hsa04727) in Dravet patient–derived lines.
**Figure S9:** KEGG mapping of the dopaminergic synapse (hsa04728) in Dravet patient–derived lines.

**Data S1:** Protein_statistic_DRAVET.

## Data Availability

The mass spectrometry proteomics data have been deposited to the ProteomeXchange Consortium via the PRIDE partner repository with the dataset identifier PXD070101. The dataset is publicly available upon publication of this manuscript. All other data supporting the findings of this study are available within the article and its Supporting Information files. Additional information may be obtained from the corresponding authors upon reasonable request.
