# Supplementary material for: Urine‐Derived iPSC Neurospheres Uncover Proteomic Correlates of Clinical Severity in Dravet Syndrome
Source: J Neurochem. 2026 Apr 29;170:e70452. doi: 10.1111/jnc.70452 (PMC13126102; doi:10.1111/jnc.70452)
Supplement: Supplementary file 1 — Table S1: Clinical severity scores from the DS‐Associated Neuropsychiatric Comorbidities Evaluation (DANCE) checklist applied to Dravet syndrome (DS) patients. Values represent composite severity scores (0%–100%; higher = worse). Individual item scores were normalized as percentages of their maximum value, then averaged within each domain to obtain domain scores. The global severity score represents the mean across all four domains. Table S2: Primer sequences. Table S3: Summary of functional module enrichment across Dravet patient‐derived neurosphere lineages and DANCE checklist. Table S4: TMT‐10plex sample assignment and batch design. Each of the two labeling batches contained nine biological samples from three Dravet syndrome iPSC‐derived neurosphere lines (DRAVET1, DRAVET2, DRAVET3; three independent differentiation replicates each) and one pooled reference sample (channel 131) used for Internal Reference Scaling (IRS) inter‐batch normalization. Figure S1: Characterization of Induced Pluripotent Stem Cells (iPSCs) generated from Dravet syndrome patients. (A) Immunofluorescence staining of the pluripotency markers OCT‐3/4, SOX2, NANOG, TRA 1‐60, TRA‐1‐81 and SSEA‐4. DAPI shows nuclei counterstaining in blue. Scale bar: 100 μm. (B) Silencing of Sendai reprogramming factors was confirmed by RT‐PCR. (C) Germ layer markers transcripts of iPSCs cells differentiated into embryoid bodies; endoderm marker alpha‐feto protein (AFP), mesoderm marker homeobox protein MSX‐1 and ectoderm marker paired box gene 6 (PAX6) by RT‐PCR. Glyceraldehyde 3 phosphate dehydrogenase (GAPDH) was used as internal control. Figure S2: Aneuploidy analysis of Induced Pluripotent Stem Cells (iPSCs) using low‐pass whole genome sequencing. Chromosome copy number analysis was carried out using low‐pass whole genome sequencing. Diagrams are a snapshot of IGV Light Whole Genome View screen depicting overviews of the cell lines diploid chromosome sets. Dots correspond to sequencing tiles approximately 2 [file JNC-170-e70452-s002.docx]

**Supplementary methods and figures**

**Urine-Derived iPSC Neurospheres Uncover Proteomic Correlates of Clinical Severity in Dravet Syndrome**

Authors: Michele Martins^1^, Guillaume Nugue^1^, Andrey Aguiar^2^, Leticia RQ Souza^2^, Paulo V. Abrantes^2, 5^, Julia Rodrigues Trajano^2^, Mariana Stelling^3^, Magno Junqueira^1*^, Stevens Rehen^2, 4*^, Marília Zaluar P. Guimarães^2, 5*^.

^1^ Departamento de Bioquímica, Instituto de Química, Universidade Federal do Rio de Janeiro, Rio de Janeiro, Brazil;

^2^ Instituto D’Or de Pesquisa e Ensino (IDOR), Rio de Janeiro, Brazil;

^3^ Instituto Federal do Rio de Janeiro, Rio de Janeiro, Brazil;

^4^ Departamento de Genética, Instituto de Biologia, Universidade Federal do Rio de Janeiro (UFRJ), Rio de Janeiro, Brazil;

^5^ Instituto de Ciências Biomédicas, Universidade Federal do Rio de Janeiro (UFRJ), Rio de Janeiro, Brazil.

*corresponding authors: Magno Junqueira magnojunqueira@iq.ufrj.br; Stevens Rehen stevens.rehen@idor.org; Marília Zaluar P. Guimarães [marilia.zaluar@idor.org](mailto:marilia.zaluar@idor.org).

## **Supplementary Table 1.** Clinical severity scores from the DS-Associated Neuropsychiatric Comorbidities Evaluation (DANCE) checklist applied to Dravet syndrome (DS) patients. Values represent composite severity scores (0–100%; higher = worse). Individual item scores were normalized as percentages of their maximum value, then averaged within each domain to obtain domain scores. The global severity score represents the mean across all four domains.

| **Domain / Variable** | **DRVT1** | **DRVT2** | **DRVT3** | **Max** |
| --- | --- | --- | --- | --- |
| **Cognition and behavior** |  |  |  |  |
| Language | 0 | 1 | 3 | 3 |
| Intelligence quotient (IQ) | 2 | 5 | 4 | 5 |
| Developmental quotient (DQ) | 0 | 2 | 2 | 2 |
| General difficulties | 4 | 10 | 9 | 15 |
| Comorbidities | 4 | 10 | 5 | 14 |
| **Domain Score** | **10/39 (26%)** | **28/39 (72%)** | **23/39 (59%)** | **39/39 (100%)** |
| **Motor abilities** |  |  |  |  |
| Mobility | 0 | 3 | 3 | 3 |
| Impaired abilities | 2 | 5 | 5 | 5 |
| **Domain Score** | **2/8**  **(25%)** | **8/8**  **(100%)** | **8/8**  **(100%)** | **8/8**  **(100%)** |
| **Daily living** |  |  |  |  |
| Self-care | 1 | 2 | 2 | 2 |
| Feeding (quantity) | 1 | 2 | 2 | 2 |
| Feeding (appetite) | 0 | 1 | 0 | 1 |
| Feeding (selectivity) | 0 | 1 | 0 | 1 |
| Swallowing problems | 0 | 1 | 0 | 1 |
| Sleep | 3 | 6 | 2 | 10 |
| **Domain Score** | **5/17**  **(29%)** | **13/17**  **(76%)** | **6/17**  **(35%)** | **17/17**  **(100%)** |
| **Family quality of life** |  |  |  |  |
| Family quality of life | 2 | 3 | 4 | 4 |
| Family suffering | 3 | 4 | 4 | 4 |
| Concerns | 28 | 56 | 11 | 70 |
| **Domain Score** | **33/78**  **(42%)** | **63/78**  **(81%)** | **19/78**  **(24%)** | **78/78**  **(100%)** |
| **Global Severity Score** | **31%** | **82%** | **55%** | **100%** |
| **Severity level* |  |  |  |  |
| *0–20% = Very mild severity* |  |  |  |  |
| *21–40% = Mild severity* |  |  |  |  |
| *41–60% = Moderate severity* |  |  |  |  |
| *61–80% = High severity* |  |  |  |  |
| *81–100% = Very high severity* |  |  |  |  |


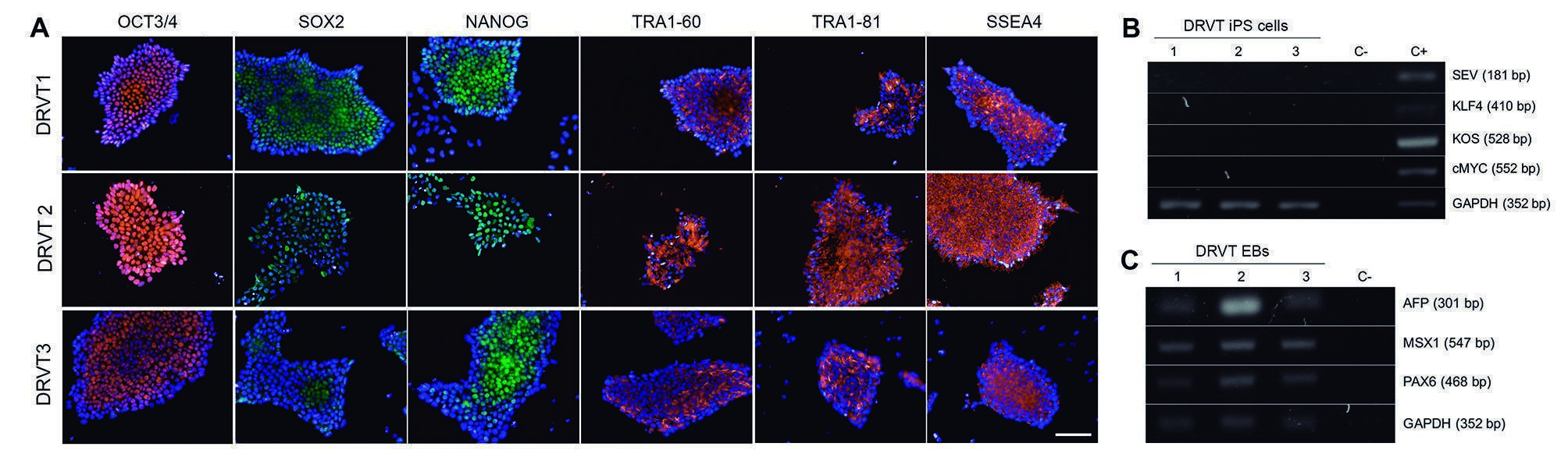


**Supplementary Figure S1**: Characterization of Induced Pluripotent Stem Cells (iPSCs) generated from Dravet syndrome patients. (A) Immunofluorescence staining of the pluripotency markers OCT-3/4, SOX2, NANOG, TRA 1-60, TRA-1-81 and SSEA-4. DAPI shows nuclei counterstaining in blue. Scale bar: 100 μm. (B) Silencing of Sendai reprogramming factors was confirmed by RT-PCR. (C) Germ layer markers transcripts of iPSCs cells differentiated into embryoid bodies; endoderm marker alpha-feto protein (AFP), mesoderm marker homeobox protein MSX-1 and ectoderm marker paired box gene 6 (PAX6) by RT-PCR. Glyceraldehyde 3 phosphate dehydrogenase (GAPDH) was used as internal control.

### **Immunocytochemistry**

Cell colonies were fixed in 4% paraformaldehyde for 15 min, permeabilized with 0.3% Triton X-100 (Sigma-Aldrich, USA) and blocked with 2% albumin (Sigma-Aldrich, USA). Immunostaining was performed using the following primary and secondary antibodies: Sox2 (MAB5603, Merck Millipore), Oct-3/4 (SC279, Santa Cruz Biotechnology), TRA1-60 (MAB4360, Merck Millipore), SSEA-4 (MAB4304, Merck Millipore), TRA1-81 (MAB4381, Merck Millipore), and Nanog (AB9220, Merck Millipore), all at 1:100 dilutions. Secondary antibodies were goat anti-rabbit Alexa Fluor 488 (A11008, Thermo Fisher Scientific) and goat anti-mouse Alexa Fluor 546 (A11032, Thermo Fisher Scientific), both at a 1:400 dilution. Images were acquired using the EVOS XL Cell Imaging System (Thermo Fisher Scientific, USA).

### **Detection of transcripts by PCR**

RNA was isolated from cultured cells using GeneJet RNA extraction kit (Thermo Fisher Scientific, USA) following the manufacturer’s instructions, and subjected to DNase treatment (DNase I, Thermo Fisher Scientific, USA). cDNA was generated from 1 µg of DNase-treated RNA using M-MLV Reverse Transcriptase (Thermo Fisher Scientific, USA). PCR reactions were performed in 10 µL volume using 5 µL of input cDNA, 1.5 mM MgCl2, 0.2 mM dNTPs, 0.2 µM of each primer and 1 U of Taq Platinum (Thermo Fisher Scientific, USA). Cycling conditions varied according to PCR product size and primers’ melting temperature, but all reactions had a common initial denaturation step of 95oC for 3 min, followed by 35 cycles of denaturation at 95oC for 15 s, annealing at primer specific temperature for 15 s, and elongating at 72oC for amplicon-specific time. All specific conditions are detailed in the Supplementary Table 1.

**Supplementary table 2: Primer sequences**

| **Primer** | **Sequence 5’-3’** | **Amplicon size** | **Annealing temp.** | **Elongation time** |
| --- | --- | --- | --- | --- |
| **SeV-F** | GGA TCA CTA GGT GAT ATC GAG C* | 181 bp | 55^o^C | 13 s |
| **SeV-R** | ACC AGA CAA GAG TTT AAG AGA TAT GTA TC* |  |  |  |
| **Klf4-F** | TTC CTG CAT GCC AGA GGA GCC C | 410 bp | 55^o^C | 25 s |
| **Klf4-R** | AAT GTA TCG AAG GTG CTC AA* |  |  |  |
| **KOS-F** | ATG CAC CGC TAC GAC GTG AGC GC | 528 bp | 55^o^C | 35 s |
| **KOS-R** | ACC TTG ACA ATC CTG ATG TGG |  |  |  |
| **cMyc-F** | TAA CTG ACT AGC AGG CTT GTC G* | 552 bp | 55^o^C | 35 s |
| **cMyc-R** | TCC ACA TAC AGT CCT GGA TGA TGA TG |  |  |  |
| **GAPDH-F** | TTC GAC AGT CAG CCG CAT C | 352 bp | 58^o^C | 13 s |
| **GAPDH-R** | GAC TCC ACG ACG TAC TCA GC |  |  |  |
| **AFP-F** | AGA GTT GCT AAA GGA TAC CAG GA | 301 bp | 50^o^C | 18 s |
| **AFP-R** | AGG CCA ATA GTT TGT CCT CAC |  |  |  |
| **MSX-F** | CCC TGG TGC TGT ACC CC | 547 bp | 50^o^C | 35 s |
| **MSX-R** | GGT CCC TTC AAC CTA CCT T |  |  |  |
| **Pax6-F** | AGA AAG AGT TTG AGA GAA CCC AT | 468 bp | 50^o^C | 28 s |
| **Pax6-R** | TCA TGT GTG TCT GCA TAT GTG G |  |  |  |
| **hcMyc-F** | AGA CAG ATC AGC AAC AAC CGA A | 469 bp | 58^o^C | 30 s |
| **hcMyc-R** | TTT GAG GCA GTT TAC ATT ATG GC |  |  |  |
| **hKlf4-F** | ATG CCA GAG GAG CCC AAG | 532 bp | 58^o^C | 35 s |
| **hKlf4-R** | AGA TCC AGT CAC AGA CCC CAT |  |  |  |
| **hNanog-F** | AAG TCT TAA AGC TGC CTT AAC CTT | 561 bp | 55^o^C | 35 s |
| **hNanog-R** | TTG CTA TTC TTC GGC CAG T |  |  |  |
| **hOct3/4-F** | CAA AAC CCG GAG GAG TCC CA | 424 bp | 58^o^C | 30 s |
| **hOct3/4-R** | CCT TCT CGA GCC CAA GCT G |  |  |  |


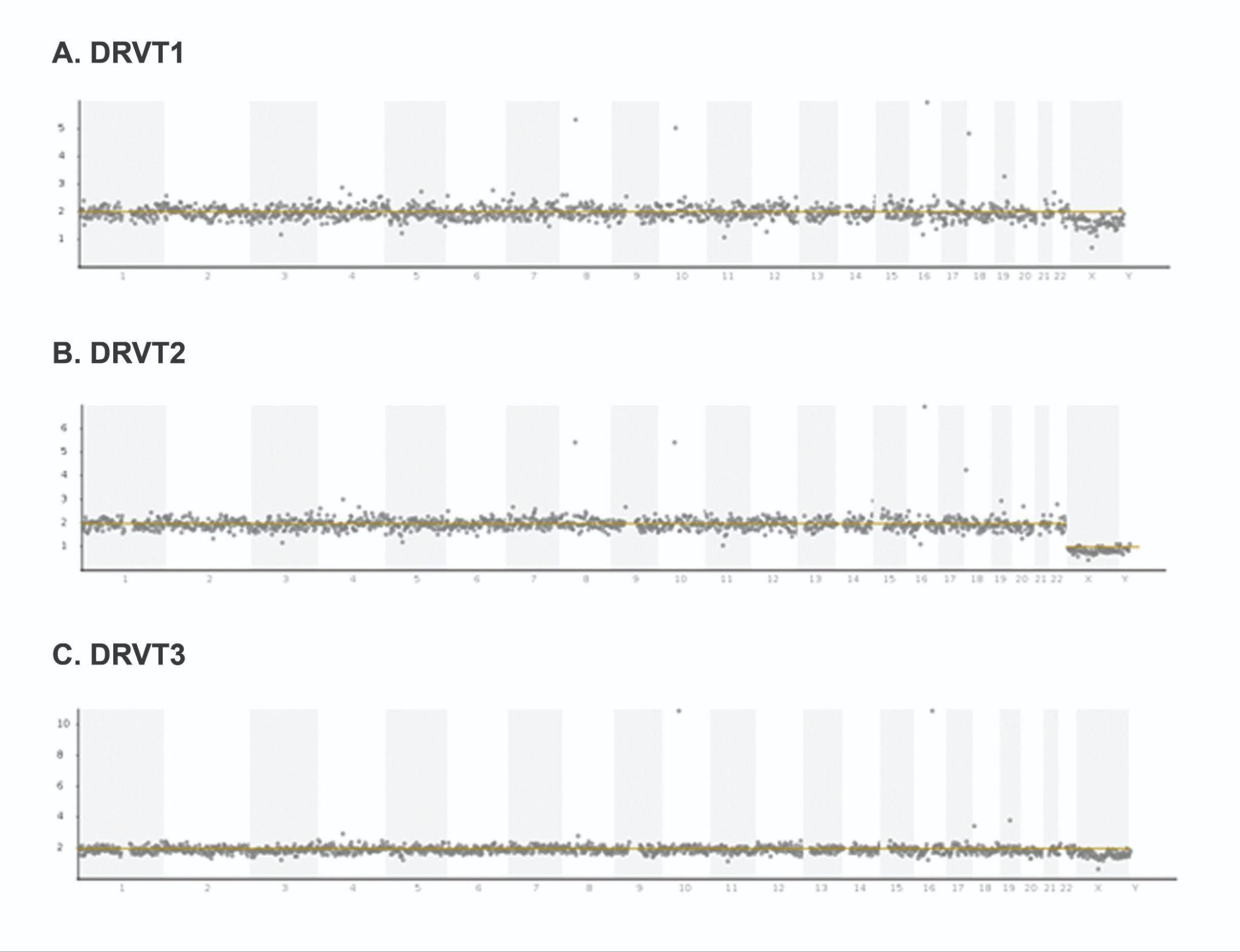


**Supplementary Figure S2**: Aneuploidy analysis of Induced Pluripotent Stem Cells (iPSCs) using low-pass whole genome sequencing. Chromosome copy number analysis was carried out using low-pass whole genome sequencing. Diagrams are a snapshot of IGV Light Whole Genome View screen depicting overviews of the cell lines diploid chromosome sets. Dots correspond to sequencing tiles approximately 2 Mb long. (A) DRVT-1: MAPD (median absolute pairwise difference): 0.129; read count: 459,214; total number of bases: 88.7 Mb; total number of bases (AQ20): 78.6 Mb; % bases (AQ20): 88.6; mean coverage depth(fold): 0.0286. (B) DRVT-2: MAPD: 0.128; read count: 604,906; total number of bases: 122 Mb; total number of bases (AQ20): 108 Mb; % bases (AQ20): 88.5; mean coverage depth(fold): 0.04. (C) DRVT-3: MAPD: 0.129; read count: 612,418; total number of bases: 113 Mb; total number of bases (AQ20): 99 Mb; % bases (AQ20): 87.6; mean coverage depth(fold): 0.0

**
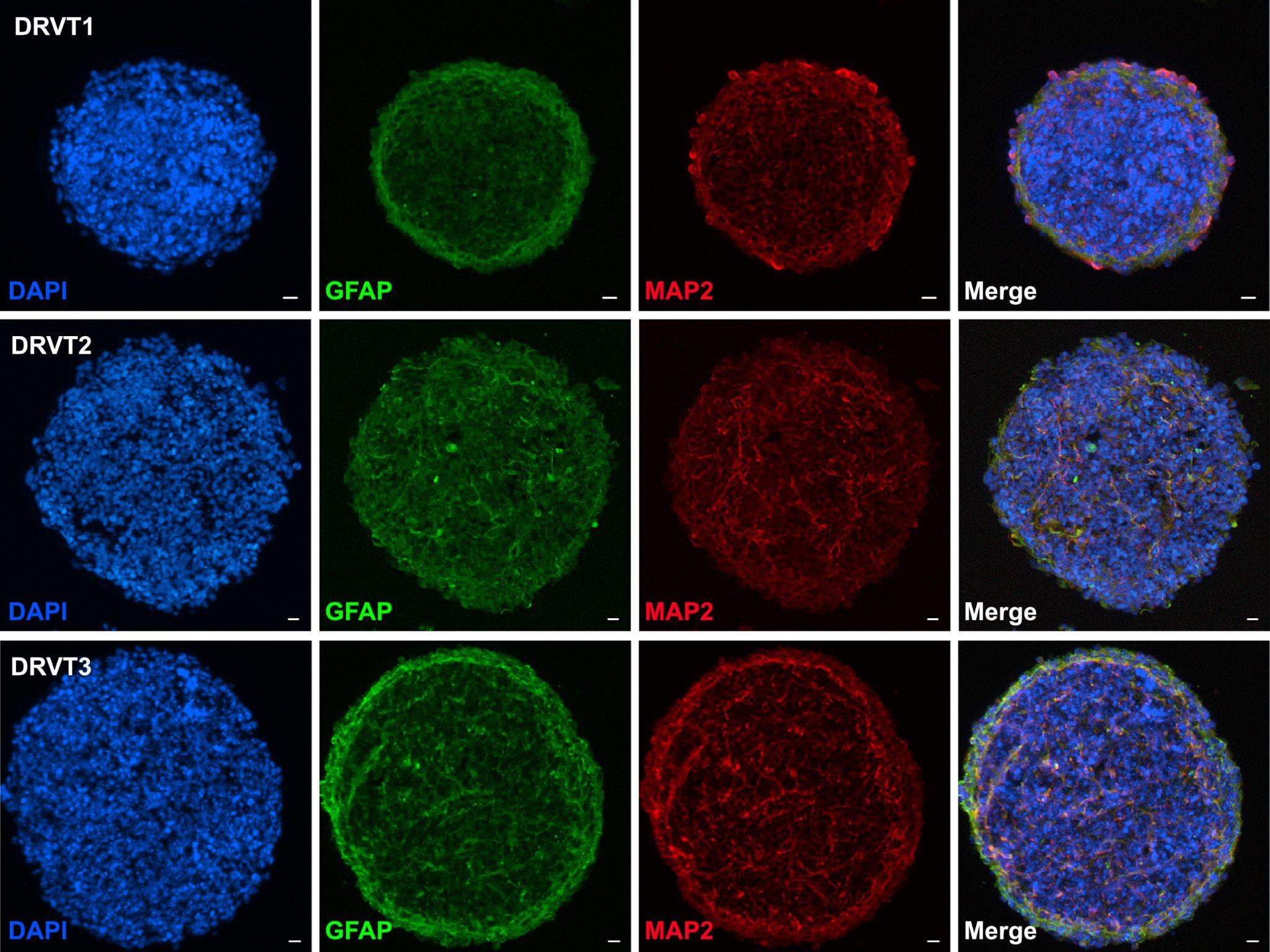
Supplementary Figure S3. Immunofluorescence detection of GFAP and MAP2 in patient-derived neurospheres.** Representative immunofluorescence images of neurospheres derived from three Dravet syndrome iPSC lines (DRVT1–3). Nuclei are labeled with DAPI (blue), GFAP (green) marks radial glia/astroglial lineage cells, and MAP2 (red) indicates neuronal differentiation. GFAP-positive cells are distributed throughout the spheroids, consistent with the presence of glial-lineage cells at early developmental stages. MAP2 signal is detectable but displays diffuse morphology without organized dendritic structures. Merged images illustrate the spatial distribution of these cell populations within the neurospheres. Scale bars: 10 µm.

**
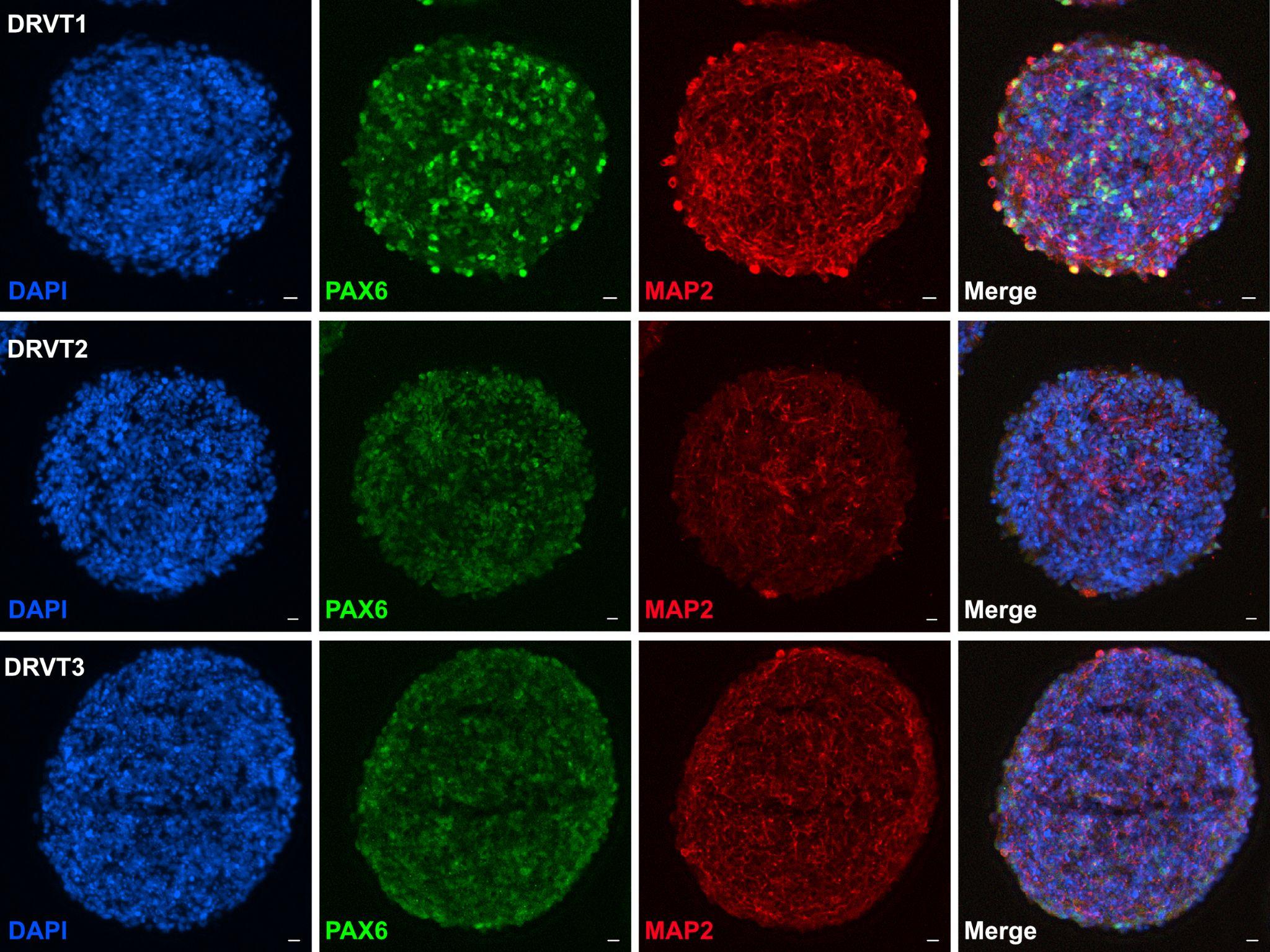
Supplementary Figure S4. Immunofluorescence detection of PAX6 and MAP2 in patient-derived neurospheres.** Representative immunofluorescence images of neurospheres derived from three Dravet syndrome iPSC lines (DRVT1–3). Nuclei are labeled with DAPI (blue), PAX6 (green) marks neural progenitor populations, and MAP2 (red) indicates neuronal differentiation. PAX6-positive cells are observed across the spheroids, supporting the persistence of neural progenitors at this stage. MAP2 expression is present but exhibits diffuse distribution, consistent with early neuronal differentiation. Merged images show co-distribution of progenitor and neuronal markers. Scale bars: 10 µm.


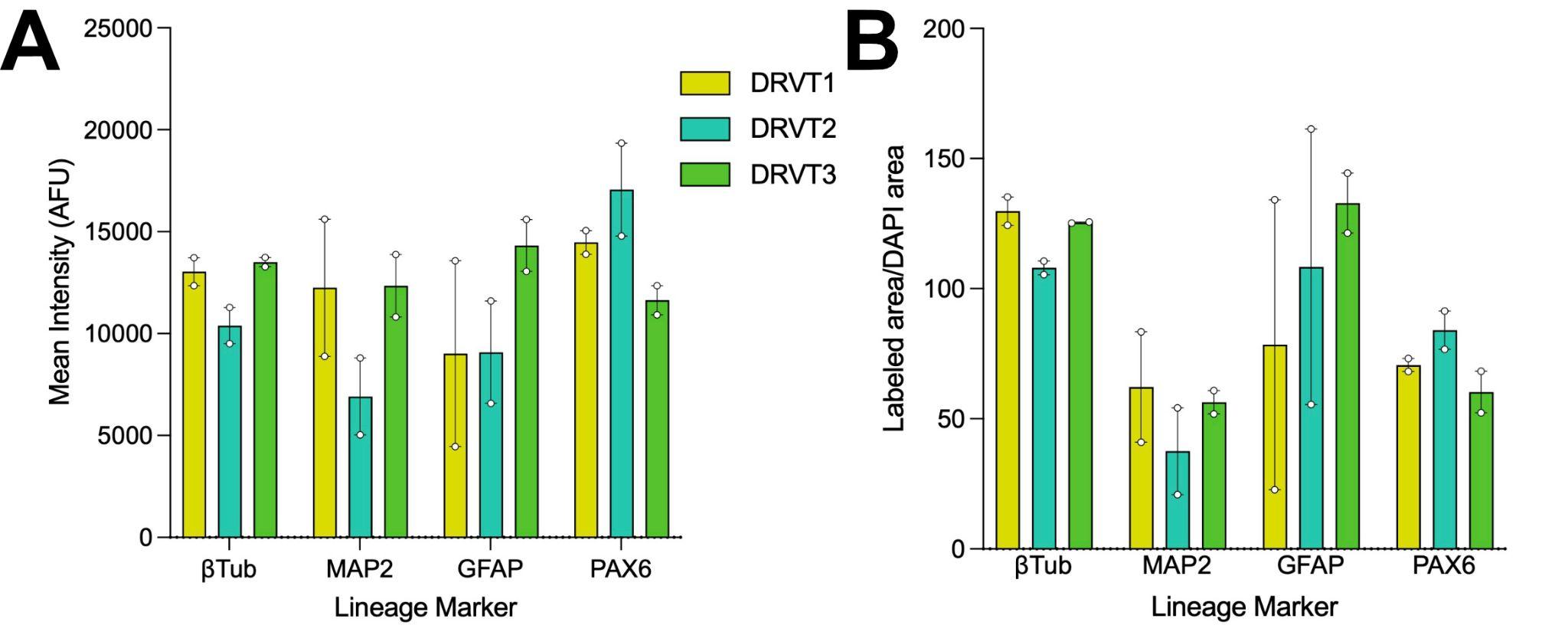


**Supplementary Figure S5. Quantification of lineage marker expression in patient-derived neurospheres.** (A) Mean fluorescence intensity (arbitrary fluorescence units, AFU) for βIII -tubulin (βTub), MAP2, GFAP, and PAX6 across neurospheres derived from three Dravet syndrome iPSC lines (DRVT1–3). Bars represent mean values for each line, and individual data points correspond to independent measurements. (B) Quantification of labeled area normalized to DAPI area for βIII-tubulin (βTub), MAP2, GFAP, and PAX6 across the same neurospheres. Bars represent mean values, and individual data points correspond to independent differentiation of neurospheres. Error bars represent SEM. For each differentiation (n=2), between 20 and 215 neurospheres were analyzed. Together, these analyses indicate the presence of neuronal (βIII-tubulin, MAP2), glial (GFAP), and progenitor (PAX6) markers across all lines, consistent with mixed neural populations at an early developmental stage.

**Supplementary Table 3**: Summary of functional module enrichment across Dravet patient-derived neurosphere lineages and DANCE checklist.

| **Processes / pathway** | **Conditions** | **Relation to DANCE** |
| --- | --- | --- |
|  |  | **(DRVT1: 29% < DRVT3: 71% < DRVT2: 88%)** |
| Synapse (SV cycle, exocytosis, organization, neurotransmitter release) | Exclusive up to DRVT1 | Consistent: preserved in the least impaired |
| Axon guidance | DRVT1 > DRVT2 | Consistent: higher in DRVT1 (mild) vs DRVT2 (severe) |
| CAMs (cell adhesion molecules) | DRVT1 > DRVT3 > DRVT2 | Consistent: follows clinical gradient |
| L1CAM interactions | DRVT1 > DRVT3/DRVT2 | Consistent: favors DRVT1 (mild) |
| Focal adhesion / Gap junction | DRVT1 > DRVT3 > DRVT2  (gap junction absent in DRVT3) | Consistent: adhesion/coupling stronger in less impaired |
| Extracellular matrix organization | DRVT3 > DRVT1/DRVT2 | Not gradient-related: DRVT3-specific structural feature |
| Collagen fibril organization | Exclusive up to DRVT3 | Not gradient-related: DRVT3-specific |
| OXPHOS / TCA / ATP / Cristae | DRVT1 ≈ DRVT3 > DRVT2 | Consistent: preserved in the less impaired |
| Cristae formation / mitochondrial matrix | DRVT1 ≈ DRVT3 > DRVT2 | Consistent: supports preserved ultrastructure in less impaired |
| RNA splicing | DRVT2 > DRVT1/DRVT3 | Consistent: enriched in the most severe |
| Cytoplasmic RNP / stress granules | DRVT2 > DRVT1/DRVT3 | Consistent: enriched in the most severe |
| Ribosome | DRVT2 > DRVT1/DRVT3 | Consistent: enriched in the most severe |
| Proteasome | DRVT2 > DRVT1/DRVT3 | Consistent: enriched in the most severe |
| Mitochondrial translation (init/elong/term) | DRVT2 > DRVT1/DRVT3 | Consistent: enriched in the most severe |
|  |  |  |


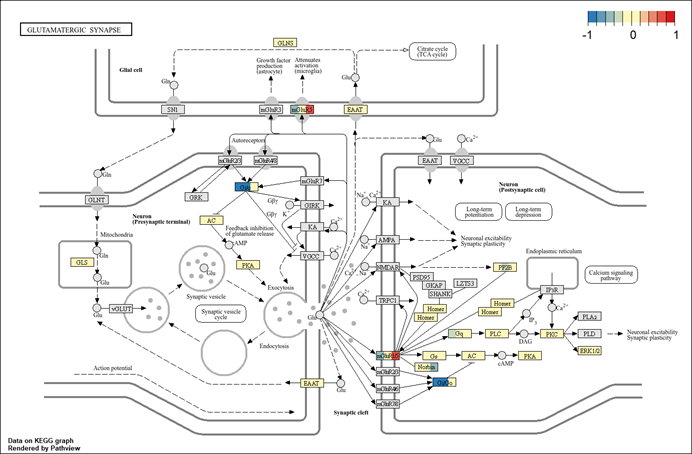


**Supplementary Figure S6**: KEGG mapping of the glutamatergic synapse (hsa04724) in Dravet patient–derived lines.

Differentially abundant proteins are overlaid onto the KEGG pathway. Proteins in yellow were identified in our dataset, and blue/red shading corresponds to Log₂ fold change values for the pairwise comparisons (DRVT2_DRVT1, DRVT3_DRVT1, DRVT3_DRVT2), as indicated by the color scale.


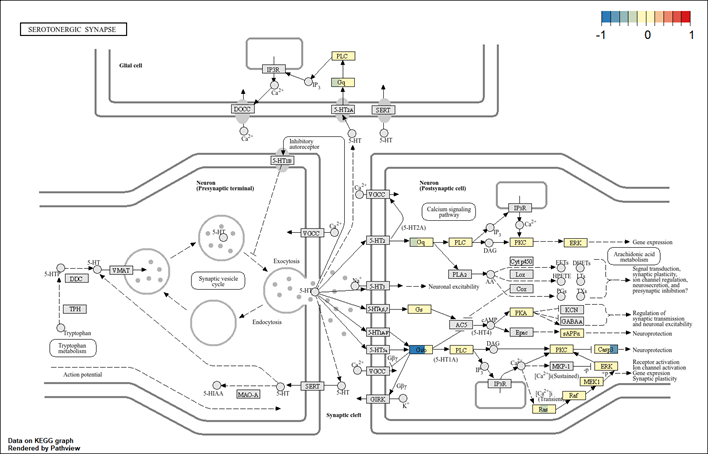


**Supplementary Figure S7**: KEGG mapping of the serotonergic synapse (hsa04726) in Dravet patient–derived lines.

Differentially abundant proteins are overlaid onto the KEGG pathway. Proteins in yellow were identified in our dataset, and blue/red shading corresponds to Log₂ fold change values for the pairwise comparisons, as indicated by the color scale.


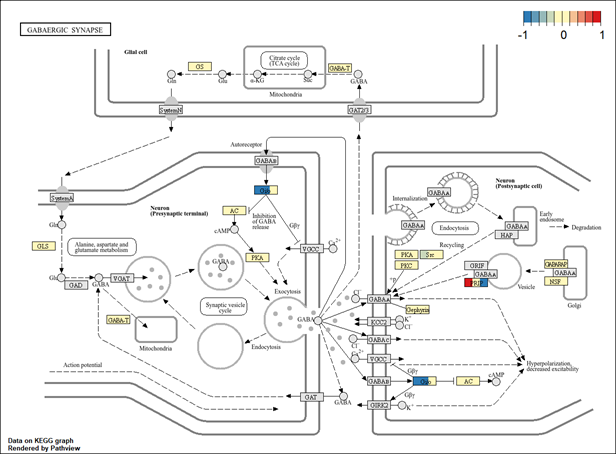


**Supplementary Figure S8**: KEGG mapping of the GABAergic synapse (hsa04727) in Dravet patient–derived lines.

Differentially abundant proteins are overlaid onto the KEGG pathway. Proteins in yellow were identified in our dataset, and blue/red shading corresponds to Log₂ fold change values for the pairwise comparisons, as indicated by the color scale.


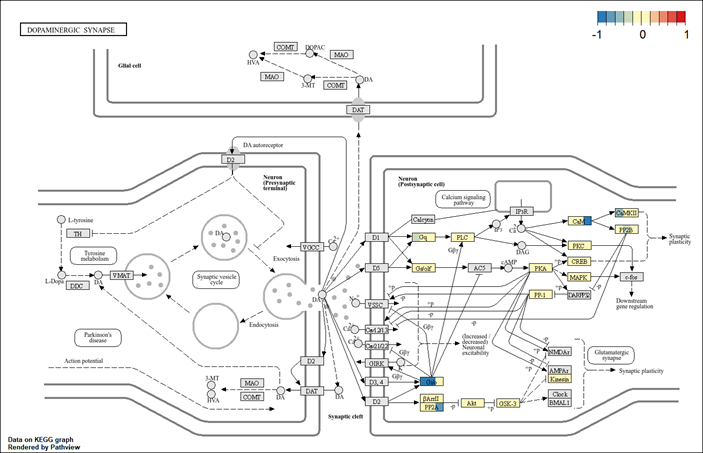


**Supplementary Figure S9**: KEGG mapping of the dopaminergic synapse (hsa04728) in Dravet patient–derived lines.

Differentially abundant proteins are overlaid onto the KEGG pathway. Proteins in yellow were identified in our dataset, and blue/red shading corresponds to Log₂ fold change values for the pairwise comparisons, as indicated by the color scale.

**Supplementary Table 4**: **TMT-10plex sample assignment and batch design.** Each of the two labeling batches contained nine biological samples from three Dravet syndrome iPSC-derived neurosphere lines (DRAVET1, DRAVET2, DRAVET3; three independent differentiation replicates each) and one pooled reference sample (channel 131) used for Internal Reference Scaling (IRS) inter-batch normalization.

| **Batch** | **TMT Channel** | **Sample ID** | **Cell Line** | **Biological Replicate** | **Sample Type** |
| --- | --- | --- | --- | --- | --- |
| 1 | 126 | DRAVET1_1 | DRAVET1 | 1 | Sample |
| 1 | 127N | DRAVET2_1 | DRAVET2 | 1 | Sample |
| 1 | 127C | DRAVET3_1 | DRAVET3 | 1 | Sample |
| 1 | 128N | DRAVET1_2 | DRAVET1 | 2 | Sample |
| 1 | 128C | DRAVET2_2 | DRAVET2 | 2 | Sample |
| 1 | 129N | DRAVET3_2 | DRAVET3 | 2 | Sample |
| 1 | 129C | DRAVET1_3 | DRAVET1 | 3 | Sample |
| 1 | 130N | DRAVET2_3 | DRAVET2 | 3 | Sample |
| 1 | 130C | DRAVET3_3 | DRAVET3 | 3 | Sample |
| 1 | 131 | POOL | POOL | N/A | Reference (IRS) |
| 2 | 126 | DRAVET1_1 | DRAVET1 | 1 | Sample |
| 2 | 127N | DRAVET2_1 | DRAVET2 | 1 | Sample |
| 2 | 127C | DRAVET3_1 | DRAVET3 | 1 | Sample |
| 2 | 128N | DRAVET1_2 | DRAVET1 | 2 | Sample |
| 2 | 128C | DRAVET2_2 | DRAVET2 | 2 | Sample |
| 2 | 129N | DRAVET3_2 | DRAVET3 | 2 | Sample |
| 2 | 129C | DRAVET1_3 | DRAVET1 | 3 | Sample |
| 2 | 130N | DRAVET2_3 | DRAVET2 | 3 | Sample |
| 2 | 130C | DRAVET3_3 | DRAVET3 | 3 | Sample |
| 2 | 131 | POOL | POOL | N/A | Reference (IRS) |
